# Cell Homeostasis or Cell Death—The Balancing Act Between Autophagy and Apoptosis Caused by Steatosis-Induced Endoplasmic Reticulum (ER) Stress

**DOI:** 10.3390/cells14060449

**Published:** 2025-03-18

**Authors:** Anna Stilkerich, Gerda Schicht, Lena Seidemann, René Hänsel, Adrian Friebel, Stefan Hoehme, Daniel Seehofer, Georg Damm

**Affiliations:** 1 Department of Hepatobiliary Surgery and Visceral Transplantation, University Hospital, Leipzig University, 04103 Leipzig, Germany; anna.stilkerich@medizin.uni-leipzig.de (A.S.); gerda.schicht@sikt.uni-leipzig.de (G.S.); lena.seidemann@medizin.uni-leipzig.de (L.S.); daniel.seehofer@medizin.uni-leipzig.de (D.S.); 2 Saxonian Incubator for Clinical Translation (SIKT), Leipzig University, 04103 Leipzig, Germany; rene.haensel@sikt.uni-leipzig.de (R.H.); friebel@izbi.uni-leipzig.de (A.F.); hoehme@izbi.uni-leipzig.de (S.H.); 3 Interdisciplinary Center for Bioinformatics (IZBI), University of Leipzig, Haertelstraße 16-18, 04107 Leipzig, Germany; 4 Center for Scalable Data Analytics and Artificial Intelligence (ScaDS.AI), 04105 Leipzig, Germany

**Keywords:** unfolded protein response, ER stress, hepatic lipid metabolism, MASLD, liver regeneration, primary human hepatocytes

## Abstract

Metabolic-dysfunction-associated steatotic liver disease (MASLD) is a prevalent liver condition with potential progression to cirrhosis and impaired regeneration post-resection. A key mechanism underlying lipotoxicity is endoplasmic reticulum (ER) stress, particularly the activation of the unfolded protein response (UPR). This study investigates the interplay between lipid accumulation, endoplasmic reticulum (ER) stress, and cellular outcomes, focusing on the balance between autophagy and apoptosis. We cultured primary human hepatocytes (PHH) in a free fatty acid (FFA)-enriched medium for 120 h, assessing lipid accumulation, metabolism, and the expression of selected UPR markers. Additionally, we investigated the effects of lipid load on cell activity and growth in proliferating HepG2 cells. We observed that FFA uptake consistently induced ER stress, shifting cellular responses toward apoptosis under high lipid loads. Donor-specific differences were evident, particularly in lipid storage, excretion, and sensitivity to lipotoxicity. Some donors exhibited limited triglyceride (TAG) storage and excretion, leading to an excess of FFA whose metabolic fate remains unclear. Proliferation was more sensitive to lipid accumulation than overall cell activity, with even low FFA concentrations impairing growth, highlighting the vulnerability of regenerative processes to steatosis. The study elucidates how ER stress pathways, such as PERK-CHOP and IRE1α-JNK, are differentially activated in response to lipid overload, tipping the balance toward apoptosis in severe cases. The limited activation of repair mechanisms, such as autophagy, further emphasizes the critical role of ER stress in determining hepatocyte fate. The donor-dependent variability highlights the need for personalized strategies to mitigate lipotoxic effects and enhance liver regeneration in steatosis-related conditions.

## 1. Introduction

With the increasing prevalence of diabetes, obesity and metabolic syndrome, the associated metabolic dysfunction-associated steatotic liver disease (MASLD), formerly known as non-alcoholic fatty liver disease (NAFLD), is becoming increasingly important in healthcare. Affecting 10–30% of the global population, it is one of the most common liver diseases and can progress to steatohepatitis, liver fibrosis, and cirrhosis [1,2]. Even mild steatosis (up to 30%) significantly increases complications after liver resection, while advanced steatosis is associated with higher postoperative mortality [3]. Although the liver has a strong regenerative capacity, obesity and high saturated fat diets reduce this ability after liver resection, as shown in animal models [4,5]. Until now, therapeutic options targeting MASLD are still based on the reduction in hepatic lipid load, either by lifestyle changes or medication targeting dyslipidemia [6]. Current research in MASLD focuses on the cellular mechanisms that are affected by lipid accumulation such as metabolism or autophagy [7,8]. Our recent work highlights endoplasmic reticulum (ER) stress as a key consequence of lipid accumulation in hepatocytes reducing their regenerative capacity [9].

It is known that there is a significant difference in the metabolism of saturated and unsaturated fatty acids. Unsaturated fatty acids, like oleate, are used for beta oxidation or triglyceride (TAG) production and stored in lipid droplets (LD) or excreted as very low-density lipoproteins (VLDL) [10]. Saturated fatty acids such as palmitate are incorporated into diacylglycerides (DAG) and, depending on the fatty acid (FA) environment, further metabolized to TAG. In the presence of unsaturated FA, palmitate is more likely directed to TAG synthesis [11]. This effect is partly due to the enzyme desaturase, which converts saturated to monounsaturated FA and is more active in an unsaturated FA-containing environment [12]. However, saturated FAs are associated with greater DAG accumulation in the ER [7] as well as increased phospholipid synthesis, leading to ER membrane dilation and stiffening and subsequent ER stress [13].

ER stress can be activated during physiological processes, such as postprandial glucose metabolism regulation. Prolonged ER stress due to MASLD is linked to inflammation and cell death and may progress to metabolic-dysfunction-associated steatohepatitis (MASH). ER stress activates the unfolded protein response (UPR), a complex signaling cascade involving multiple pathways and feedback loops that affect transcription, autophagy, protein processing, and apoptosis [14]. It is initiated by three transmembrane proteins at the ER, bound to BiP (binding immunoglobulin protein): inositol requiring enzyme 1α (IRE 1α), protein kinase RNA-activated-like ER kinase (PERK) and activating transcription factor 6α (ATF 6α). Upon activation, ATF 6α is cleaved inside the Golgi apparatus; afterward, its N-terminal fragment enters the nucleus to activate peroxisome-proliferator-activated receptor α (PPARα), a transcription factor regulating FA oxidation [15]. The activation of IRE 1α leads to phosphorylation of Janus kinase (JNK), which can trigger apoptosis through the activation of further signal proteins [16]. Additionally, IRE 1α and JNK are linked to autophagy involving microtubule-associated protein 1 light chain 3β (MAP-LC3β) [17]. PERK activation inhibits mRNA translation and, through several intermediates, activates CHOP (transcriptional factor C/EBP homologous protein), a transcription factor that induces apoptosis [16]. The pathway to apoptosis involves the cleavage of PARP (Poly (ADP-ribose) polymerase) [18]. The balance between autophagy and apoptosis is tightly regulated and decides between the life and death of the cell [19].

Current knowledge on the pathophysiological link between ER stress and MASLD is mainly based on studies in animals and cell lines. Evidence from human liver tissues or primary hepatocytes is scarce. Kuo et al. used human hepatocytes from a commercial source and analyzed steatosis-induced apoptosis, focusing on the PERK-transglutaminase 2 pathway [20]. Our study extends recent studies such as the work of Kuo et al. by using primary human hepatocytes (PHH) from multiple donors with well-characterized clinical backgrounds, enabling us to investigate donor-specific differences in lipid metabolism and ER stress. Additionally, we explored a broader range of pathways, including autophagy, apoptosis, and the role of lipid accumulation, highlighting novel insights into the regenerative impairment and variability in MASLD progression.

Our study aimed to investigate the relationship between hepatocellular lipid processing and UPR activation and its downstream mechanisms in humans. We utilized a well-established human steatosis model based on PHH. PHH were cultured with a mixture of free fatty acids (FFA) for up to 120 h, during which we measured fatty acid uptake, lipid accumulation and excretion, and the expression of key UPR proteins and their downstream targets. Additionally, we examined the effects of lipid uptake on cell growth and activity using a proliferating HepG2 model. Our findings provide insights into the transition from intracellular lipid homeostasis to pathological lipotoxicity in steatosis. We also demonstrate that even low concentrations of FFA can impair cell proliferation and the regenerative capacity of the human liver.

## 2. Materials and Methods

### 2.1. Liver Tissue Samples, Isolation, and the Cultivation of Primary Human Hepatocytes

Cells from four patients were included in this study. All of them were treated in the Department of Hepatobiliary Surgery and Visceral Transplantation at University Hospital Leipzig, Germany because of benign or malign liver tumors (Table 1). The study protocol was developed according to the Declaration of Helsinki and approved by the Ethical Committee at the Medical Faculty, Leipzig University (006/17-ek, date 21 March 2017 ratified 12 February 2019). All patients gave their signed consent to participate in the study prior to surgery.

Primary human hepatocytes (PHH) were isolated from tumor-free tissue samples we obtained from resected livers. A two-step collagenase perfusion technique was used according to the protocol described earlier [21,22,23]. After washing with phosphate-buffered saline (PBS, Thermo Fisher Scientific, Waltham, MA, USA), cells were resuspended in PHH culture medium (William’s Medium E (WME), Penicillin/Streptomycin (100 U/100 µM), 15 mM HEPES (4-(2-hydroxyethyl)-1-piperazineethanesulfonic acid), 1 mM Sodium Pyruvat, 1% NEAA (Non-Essential Amino Acids Solution) (all purchased from Thermo Fisher Scientific, Waltham, MA, USA), 10% FBS (fetal bovine serum, Biochrom GmbH, Berlin, Germany), 40 U/mL Human Insulin (Elli Lilly and Company, Indianapolis, IN, USA), 1 µg/mL Dexamethason (Jenapharm GmbH + Co. KG, Jena, Germany). Cell count and viability were determined by trypan blue (Sigma Aldrich, St. Louis, MO, USA) exclusion and examined with a Neubauer chamber. Cell culture plastics (Greiner Bio-One GmbH, Frickenhausen, Germany) and µ-Slides (Ibidi GmbH, Gräfelfing, Germany) were covered with rat tail collagen that was prepared in our laboratory based on the protocol of Rajan et al. [24] Afterward, cells were seeded and attached overnight.

### 2.2. Induction of Steatosis

Based on the model of Gomez-Lechón et al. [25], steatosis was induced by a culture medium containing 0.6 mM mixture of palmitic and oleic acids (WME, 5% FBS, 15 mM HEPES, 1 mM Sodium pyruvate, 1% MEM NEAA, 100 U/100 µM Penicillin/Streptomycin, 0.2 mM Sodium-Palmitat, 0.4 mM Oleic Acid (both purchased from Sigma Aldrich (St. Louis, MO, USA)), 0.3% Methanol (Th. Geyer GmbH & Co. KG, Renningen, Germany). After adherence, cells were washed and the medium was replaced by FFA-containing medium or control medium (WME, 5% FBS, 15 mM HEPES, 1 mM Sodium pyruvate, 1% MEM NEAA, 100 U/100 µM Penicillin/Streptomycin, 0.3% Methanol). Cells were cultivated over a period of 120 h. The culture medium was changed every 24 h, and each day, samples were collected for further examination.

### 2.3. Oil Red O and SRB Staining

For Oil Red O and SRB staining, cells were washed with PBS and fixed with 4% formaldehyde (Carl Roth, Karlsruhe, Germany) solution for 10 min.

For the assay procedure, Oil Red O (Sigma Aldrich, St. Louis, MO, USA) was dissolved in isopropanol (Carl Roth, Karlsruhe, Germany) to a concentration of 0.35%, mixed with water at a ratio of 2:3, and added to fixed cells. After 20 min of incubation time, cells were washed with dH_2_O and examined with an inverse microscope (Eclipse TS100, Nikon, Tokio, Japan) for the visual detection of steatosis. Afterward, cells were dried, and dye was dissolved in isopropanol. Oil Red O concentration was detected by measuring absorbance at 492 nm in a microplate reader (Synergy H1, BioTek, Winooski, VT, USA). The remaining supernatant was removed from cells and cells were washed again with dH_2_O.

A sulforhodamine b sodium salt (SRB, Sigma Aldrich, St. Louis, MO, USA) solution was prepared by dissolving SRB in 1% acetic acid up to a concentration of 0.4% and added to cells. After an incubation time of 30 min in the dark, cells were washed four times with 1% acetic acid solution. The attached SRB was resuspended in TRIS-solution containing 10 mM TRIS (tris(hydroxymethyl)aminomethane, Carl Roth, Karlsruhe, Germany) in dH_2_O. Detection was performed via a microplate reader measuring absorbance at 565 nm separated from background staining at 690 nm.

### 2.4. Confluence Measuring

For the measuring of confluence at 0 h, microscopic pictures were taken with an inverse microscope. Three pictures per donor were examined by marking cell-covered departments manually and detecting area size by analysis with Image J (Fiji 2.16).

### 2.5. Fluorescent Staining, Microscopy, and Bioinformatic Evaluation

Cells were washed with PBS and fixed with 4% formaldehyde solution for 10 min. BODIPY™ 493/503 (Thermo Fisher Scientific, Waltham, MA, USA) was diluted in PBS to a concentration of 25 µg/mL and incubated over 45 min at room temperature in the dark. Hoechst 33,342 (Thermo Fisher Scientific, Waltham, MA, USA) was used at a concentration of 10 µg/mL in PBS with 10 min incubation time at room temperature. Phalloidin iFluor 555 (abcam, Cambridge, UK) was diluted in 1% BSA/PBS up to a concentration of 1 µL/mL and incubated at 4 °C overnight. Fluorescent detection was performed with a laser scanning microscope (LSM 700; Carl Zeiss, Oberkochen, Germany) in z-stack recordings with a thickness of 0.39 µm. In total, 188 3D confocal image stacks were acquired across 38 groups with five replicates per group (for exceptions and further details, see Supplement Table S1). Of these, three images were found to be defective due to a malfunction during imaging. Additionally, we observed a gradual x-y drift in 45 images, which was corrected using rigid body registration with StackReg [26]. Cell segmentation was performed considering the nuclei and cell membrane signals using a deep-learning-driven algorithm by Cellpose [27]. To adapt the pre-trained cyto3 model to our data, we interactively re-trained it on representative 2D XY slices [28]. A model, trained on images from diverse conditions, was applied using the Cellpose 2.5D segmentation approach, where flows of XY, ZY, and ZX slices are averaged. With this approach, 107 images were successfully segmented using two different parameter sets. For the remaining images, mostly from later time points, segmentation results were unsatisfactory due to more complex 3D shapes. In order to achieve at least three segmented images per condition, cell borders were marked manually in five stacks, and cell volume was interpolated with 3D slicer, version 5.8.0 [29,30]. All images were preprocessed with deconvolution software Huygens Professional version (24.10.0p3 64b) to recover images from the point spread function [31].

Areas outside the labeled cells were removed from the input file using the ROI mask. Background noise was eliminated by applying a median filter (sigma factor 5px) to the original image. The image was binarized using the maximum entropy threshold method, assigning white to LD pixels and black to the background. A label map was created through connected component analysis to assign LDs to individual cells. Volume was determined by pixel counting. Analysis and chart design were performed with the software Python version 3.12.9 and the seaborn statistical data visualization module.

### 2.6. LDH Assay

Supernatants were collected at their respective time points, centrifuged to remove cell debris, and stored at −80 °C. LDH activity was examined with LDH-L Assay Kit following the manufacturer’s instructions. Photometric measuring was performed in a microplate reader at 340 nm over 15 min. In donor 4 we measured negative LDH values in the FFA-treated group. For the normalization of protein expression on LDH, we adjusted these negative values to a baseline of 8.37 U/L, as they cannot be explained biologically.

### 2.7. FFA Assay

A Free Fatty Acid Assay Kit (Sigma Aldrich, St. Louis, MO, USA) was used to detect the content of the remaining FFA in supernatants. Based on the initially added free fatty acid content, we calculated the uptake within 24 h and summed them up over the whole incubation time. This assay works by activating free fatty acids via Acetyl Coenzyme A Synthetase and measuring fluorescent signal after further steps of enzyme-coupled activation that are induced by adding a reaction mix. The procedure was performed following the manufacturer’s instructions, with the exception of steatosis medium (without fatty acids and methanol) being used instead of assay buffer for dilution of standards and samples. Fluorescence was measured at 570 nm using a microplate reader.

### 2.8. TAG Assay

For the quantitative detection of TAG content and TAG excretion, samples of cell lysate and supernatants were analyzed with Triglyceride Quantification Assay Kit (abcam, Cambridge, UK). After supernatants were retained, 4% NP40 (rergitol NP 40 Cell Lysis Buffer, Sigma Aldrich (St. Louis, MO, USA)) was added and cells were scraped off. For lysis, cells were heated up to 100 °C two times for 2 min. Samples were stored at −80 °C until measurement. The kit was used according to the manufacturer’s instructions. TAG Assay works by dividing TAG to fatty acids and glycerol with Lipase, oxidizing glycerol and inducing a reaction of oxidized glycerol with a dye. Fluorescence was measured at Ex/Em 535/587 nm in a microplate reader.

### 2.9. HepG2 Cell Cultivation

HepG2 cells (HB-8065, AATC, Manassas, VA, USA) were cultured on uncoated culture plastics with HepG2 culture medium (DMEM (Dulbecco’s Modified Eagle Medium from Thermo Fisher Scientific (Waltham, MA, USA), 10% FBS, 100 U/100 μM penicillin/streptomycin). The medium was changed three times a week and after reaching a density of 70–80% cells were divided. For cell count and re-seeding, cells were detached with trypsin-EDTA (0.25%/0.02%; Biochrom GmbH, Berlin, Germany), resuspended in culture medium, washed with PBS, and counted in a Neubauer chamber after trypan blue staining.

### 2.10. Induction of UPR in HepG2 Cells Using Thapsigargin and Tunicamycin

Positive controls for Western blot analysis were produced by inducing UPR in HepG2 cells with thapsigargin and tunicamycin (both purchased from Sigma Aldrich, St. Louis, MO, USA). HepG2 cells were incubated in a culture medium containing different concentrations of tunicamycin or thapsigargin over different periods of time based on the procedures described elsewhere [32,33].

### 2.11. Induction of Steatosis in HepG2 Cells

HepG2 cells were seeded on cell culture plates and allowed to adhere overnight. A fatty acid mix containing palmitic and oleic acids in a ratio of 1:2 was prepared. HepG2 stock medium (DMEM, 5% FBS, 100 U/100 μM penicillin/streptomycin) was mixed with different concentrations of fatty acid mix (0 mM, 0.3 mM, 0.6 mM, 0.9 mM, 1.2 mM, and 1.5 mM). After adherence, the medium was changed. Cells were cultured for 48 h, and the medium was changed every 24 h.

### 2.12. Western Blot Analysis

For Western blot analysis, one part of cells were treated with radioimmunoprecipitation assay (RIPA) lysing buffer containing 50 mM Tris-hydrochloride (Carl Roth, Karlsruhe, Germany), 150 mM sodium chloride (NaCl), 1 mM ethylenediaminetetraacetic acid (EDTA), 0.5% sodium deoxycholate, 0.5 mM sodium orthovanadate, 0.4% aprotinin, 0.4% AEBSF (4-(2-aminoethyl)-benzolsulfonylfluorid hydrochlorid) (all from Sigma Aldrich (St. Louis, MO, USA)), 0.05% NP40, and 2.5 mM sodium fluoride (Honeywell, Seelze, Germany). Another part was lysed by Subcellular Fractionation Kit (Thermo Fisher Scientific, Waltham, MA, USA) following manufacturer’s instructions to analyze PPARα expression in the nucleus. Samples were stored at −80 °C.

To determine the protein concentration of the samples, BCA assay was performed. A serial dilution of bovine serum albumin (BSA) solution was used as standard. A reaction mix consisting of Bicinchoninic Acid solution (BCA) reagent A and 4% Copper(II) sulfate (CuSO_4_) (all purchased from Sigma Aldrich, St. Louis, MO, USA) was added to the standard and samples according to the manufacturer’s instructions. Absorbance was measured at 550 nm using a microplate reader.

Protein lysates were adjusted to a volume of 15–20 µL containing a definite protein amount between 17 to 25 µg (see Supplement Table S2 with RIPA buffer and 4× Laemmli sample buffer (from Bio-Rad Laboratories, Hercules, CA, USA) and heated at 95 °C for 5 min. After cooling down, samples were used immediately or stored at −80 °C for later use. Resolving gels for separating with sodium dodecyl sulfate–polyacrylamide gel electrophoresis (SDS PAGE) were prepared with a mixture of Rotiphorese Gel A and B (Carl Roth, Karlsruhe, Germany) and 0.5 M TRIS Solution, 0.1% Sodiumdodecylsulfat (SDS, Carl Roth (Karlsruhe, Germany) and 0.1% APS. Stacking gels of 5% were prepared with Rotiphorese Gel A and B and 0.5 M Tris-hydrochloride. Proteins and a protein ladder (PageRuler Prestained Protein Ladder (Thermo Fisher Scientific, Waltham, MA, USA) or Chameleon Duo Pre-stained Protein Ladder (LI-COR Biosciences, Lincoln, NE, USA)) were separated with SDS-PAGE in an electrophoresis chamber (Bio–Rad Laboratories, Hercules, CA, USA) using running buffer (2.5 mM TRIS, 19.2 mM glycine (Carl Roth, Karlsruhe, Germany), 0.1% SDS) for 30 min at 80 V to run through stacking gel and 120 V for 120 min to run through resolving gel. Afterward, gels and Odyssey Nitrocellulose Membrane (LI-COR Biosciences, Lincoln, NE, USA) were activated in blotting buffer (4.8 mM TRIS, 3.9 mM glycine, 20% methanol) for 5 min. Blotting was performed using a tank blot system (Bio–Rad Laboratories, Hercules, CA, USA) at 4 °C and 35 V for 17 h. For total protein detection, membranes were stained with Revert™ 700 Total Protein Stain (LI-COR Biosciences, Lincoln, NE, USA) for 5 min and washed two times with Revert Wash Solution (6.7% acetic acid, 30% methanol). Total protein was detected at 700 nm via Odyssey 9120 Imaging System (LI-COR Biosciences, Lincoln, NE, USA). The staining was removed by incubation in Revert Reversal Solution (0.1 M NaOH (Sodium hydroxide, Carl Roth, Karlsruhe, Germany), 30% methanol) for 5 min, and membranes were activated for 1 h in Intercept^®^ (TBS) Blocking Buffer (LI-COR Biosciences, Lincoln, NE, USA).

Primary Antibodies (Supplement Table S2) were diluted in Intercept TM (TBS) blocking buffer and 0.1% TWEEN^®^ 20 (Sigma Aldrich, St. Louis, MO, USA) in a fitting dilution and incubated at 4 °C overnight. Secondary antibody was diluted 1:10,000 in Intercept TM(TBS) blocking buffer and 0.1% TWEEN 20. Membranes were stained with secondary antibody (IRDye800CW Goat anti-rabbit, IRDye680RD Goat anti-mouse, both purchased from LI-COR Biosciences (Lincoln, NE, USA)) for 1 h at room temperature in the dark. Target protein detection was performed at 700 nm (anti-mouse) or 800 nm (anti-rabbit) with the Odyssey 9120 Imaging System. Some membranes showed a high background staining disturbing the detection of target proteins. In such cases, membranes were treated with stripping buffer (LI-COR Biosciences, Lincoln, NE, USA) according to the manufacturer’s instructions, and the incubation of antibodies was repeated. To ensure comparability, all measured signals were normalized to positive controls. For analysis, Image Studio™ Software (version 5.2, LI-COR Biosciences, Lincoln, NE, USA) was used.

### 2.13. Alamar Blue Assay

Alamar Blue Assay (Bio-Rad Laboratories, Hercules, CA, USA) solution was added to HepG2 starving medium (DMEM, 100 U/100 μM penicillin/streptomycin) at a dilution of 1:10. Culture medium was removed from cells and the Alamar Blue mixture was added. After an incubation time of 4 h at 37 °C, fluorescence was detected using a microplate reader at Ex/Em 560/590 nm.

### 2.14. Sybr Green Assay

For the SYBR Green Nucleic acid gel stain (Thermo Fisher Scientific, Waltham, MA, USA) assay, cell culture plates were washed and frozen at −20 °C overnight. Sybr Green working solution (PBS, 0.1% Triton × 100 (Sigma Aldrich, St. Louis, MO, USA), 0.04% Sybr Green) was added to the cells and incubated for 1 h at room temperature in the dark. Fluorescence intensity was detected using a microplate reader at Ex/Em 485/535 nm.

### 2.15. Statistical Analysis

Data are expressed as means + SD. Data from PHH are single values or different counts of technical replicates, as indicated in the figures. ROUT test at Q = 1% was used to identify outliers for data sets containing eight to twelve technical replicates (Oil Red O and SRB detection). Steatosis in proliferating cells was investigated in three biological replicates of HepG2 cells including 10 technical replicates each. Statistical analysis and chart design were performed with GraphPad Prism 7 software (San Diego, CA, USA). To compare multiple groups, one-way or two-way ANOVA tests followed by Tukey’s tests were performed. Statistical significance was considered at *p* < 0.05.

## 3. Results

### 3.1. Characterization of Steatotic PHH Reveals Donor-Dependent Variation

**Figure 1:**
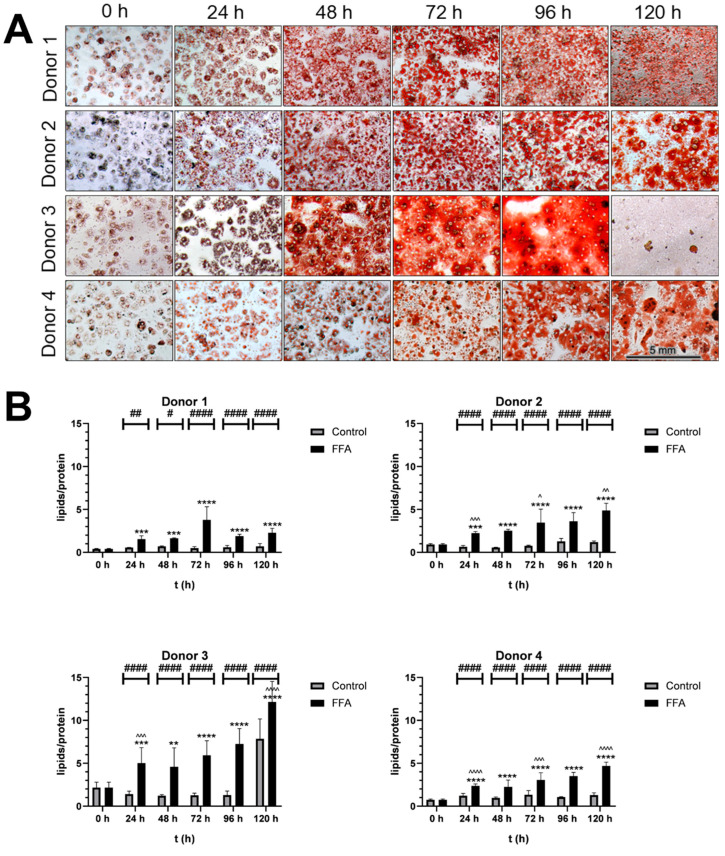
Qualitative and semi-quantitative analysis of steatosis. (**A**). Microscopic analysis of Oil Red O-stained cultures: PHH were cultivated with FFA-containing or control medium. For the qualitative analysis lipids were stained with Oil Red O and SRB. Images were taken with an inverse microscope; 20× magnification. PHH from all donors showed a visible increase in lipid load. (**B**). Semiquantitative analysis of lipids per protein: Oil Red O (ORO) extinction per SRB staining extinction: PHH were cultivated with 0.6 mM FFA or control medium. For the semiquantitative analysis of lipids per protein, ORO and SRB stainings were performed, and extinction was measured to determine relative concentrations. Data are shown as means + SD, n = 8 (donor 1) or n = 12 (donor 2, 3 and 4). Two-way ANOVA and post hoc Tukey’s test; FFA-treated and control group were compared at the same time points (####: *p* < 0.0001, ##: *p* < 0.01, #: *p* = 0.0186) and the increase in the FFA-treated group compared to the start (****: *p* < 0.0001, ***: *p* < 0.001, **: *p* = 0.0046). Further, the increase during 24 h of FFA-incubation was analyzed (^^^^: *p* < 0.0001, ^^^: *p* < 0.001, ^^: *p* < 0.01, ^: *p* < 0.05).

PHH were isolated from four human liver tissue samples (Table 1), and lipid content was assessed initially and post FFA treatment at 24 h intervals using microscopy and Oil Red O staining for the visualization of neutral lipids (Figure 1). The initial cultures showed homogenous PHH distribution with ~60% confluence (Supplement Figure S1), but lipid presence varied widely across cells, indicating baseline heterogeneity in steatosis.

Treatment with 0.6 mM FFA every 24 h for five days induced further steatosis. Figure 1A shows the time-dependent neutral lipid accumulation observed in PHH cultures from all four donors. As PHHs were cultured, confluence increased, and cells formed additional cell–cell contacts. In the control cultures, cell surface protein levels, which were detected with SRB-staining, remained stable, indicating that the cultures were well maintained over time (Supplement Figure S2). In contrast, FFA treatment led to a time-dependent decrease in protein levels, alongside an increase in lipid droplet number and size. Over time, individual lipid droplets merged, changing from a heterogenous distribution of lipid droplets to a more homogenous area of lipid content. Donor-specific differences were noted: lipid merging began at 48 h in donor 3, 72 h in donor 1 and 2, and 96 h in donor 4. After 120 h of FFA treatment, donor 3 exhibited cell loss and elevated lipotoxicity, confirmed by LDH measurements (Supplement Figure S3).

The quantification of the lipid accumulation demonstrated variability in the dynamics and capacities for lipid storage across donors (Figure 1B). Donor 1 had the lowest initial lipid-to-protein ratio, while donor 3’s ratio was 5.3 times higher. All FFA-treated cultures showed significantly increased lipid content with varying dynamics of accumulation. In the first 24 h, lipid content increased by 2.3- to 3.8-fold. Further FFA treatment caused continued lipid accumulation in donors 2, 3, and 4, while donor 1 showed a slower increase. By 120 h, lipid content had risen 5.15- to 6.34-fold in all donors.

### 3.2. Steatosis Leads to Adaptations in Hepatic Lipid Metabolism

**Figure 2:**
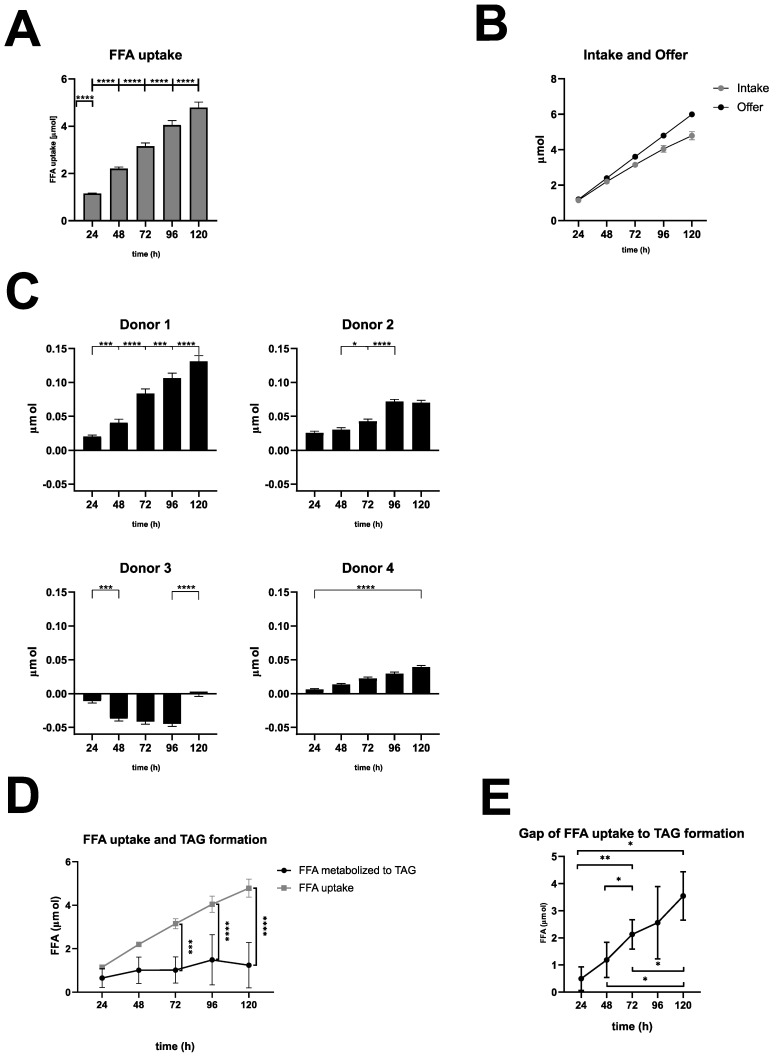
Quantitative analysis of FFA uptake, TAG excretion, and FFA metabolization. (**A**). FFA uptake: FFA uptake was calculated as the difference between initial and measured FFA concentrations in 2 mL of medium per well, summed over the entire incubation period. Data are presented as mean + SD (n = 4 biological replicates). Statistical analysis was performed using one-way ANOVA with Tukey’s post hoc test, and a one-sample t-test was used to compare the 24 h value to a hypothetical baseline (0 mM). **** *p* < 0.0001. (**B**). Comparison of FFA intake and FFA availability. (**C**). TAG excretion: TAG excretion was calculated as the difference in TAG concentrations between FFA-treated and control supernatants in 2 mL of medium per well, summed over the entire incubation period. Data are presented as mean + SD (n = 2 technical replicates). The statistical significance of TAG excretion at 24 h intervals (**** *p* < 0.0001, *** *p* < 0.001, * *p* < 0.05) or 120 h interval was determined using one-way ANOVA with Tukey’s post hoc test. (**D**). FFA uptake and TAG formation: FFA uptake compared with FFA used for TAG formation (LD and VLDL). Data are shown as mean ± SD (n = 4 biological replicates), with statistical analysis performed using two-way ANOVA and Tukey’s post hoc test (**** *p* < 0.0001, *** *p* < 0.001). (**E**). The gap of FFA uptake to TAG formation (LD and VLDL): The gap between FFA uptake and TAG formation (LD and VLDL) was calculated over time (see Table S3 in the Supplement). Data are shown as mean ± SD (n = 4 biological replicates). Statistical significance between time points was determined using one-way ANOVA with Tukey’s post hoc test (** *p* < 0.01, * *p* < 0.05).

In the next step, we investigated changes in FFA uptake, lipid metabolism, and excretion under steatotic conditions (Figure 2). Therefore, we measured FFA and TAG concentrations in our cultures using biochemical assays.

Measurements of FFA uptake revealed that the uptake of FFA into the hepatocytes was very similar in all donors. This allowed the data to be averaged (Figure 2A). We observed a significant increase in FFA uptake over the entire treatment period, with the largest increase occurring during the first 24 h (95.9% uptake), followed by a gradual flattening over time, culminating in 79.8% uptake over 120 h across all donors (Figure 2B). These results indicate that hepatocytes incorporate a major part of the offered FFA, even under severe steatotic conditions.

Excreted TAG is a surrogate for VLDL secretion and was measured as a cumulated amount of TAG in the supernatants of FFA-treated and control cultures. Results were plotted as differences between FFA-treated and control cultures (Figure 2C). Our results revealed that FFA-treated hepatocytes from donor 1 had the highest excretion rate of TAG, showing a significant increase every 24 h. Donors 2 and 4 showed a similar pattern, although with quantitatively lower TAG excretion. Notably, donor 2 seemed to limit TAG excretion after 96 h. The excretion kinetics of PHH from donor 3 varied completely in comparison to the other donors. Here, we observed a lower TAG excretion of the FFA-treated group compared to the control group resulting in negative values, except for the 120 h time point, which showed a slightly increased excretion.

In a further step, we wanted to investigate the metabolization from FFA to TAG. This includes storage TAG like LD as well as secreted TAG like VLDL (Supplement Table S3 and Supplement Figure S4). Our calculations revealed that TAG storage and excretion varied in the different donors with an increasing variation at later time points of the cultivation time. The comparison of FFA uptake and TAG formation showed a significant difference beyond 48 h (Figure 2D), resulting in an increasing gap between FFA uptake and TAG formation (Figure 2E). These observations indicate that with increasing intracellular lipid accumulation, a smaller amount of FFA are transformed into TAG and have to be utilized elsewhere.

Taken together, under steatotic conditions, hepatocytes exhibited efficient FFA uptake, particularly within the first 24 h, indicating an active response to lipid availability. TAG storage and excretion showed donor-dependent variability: while most donors demonstrated significant TAG secretion, donor 3 exhibited impaired excretion under FFA treatment. Over time, lipid accumulation resulted in a growing gap between FFA uptake and TAG formation, suggesting that hepatocytes increasingly diverted FFA to alternative metabolic pathways as TAG synthesis became saturated.

### 3.3. Investigation of Lipid Droplet Dynamics

**Figure 3:**
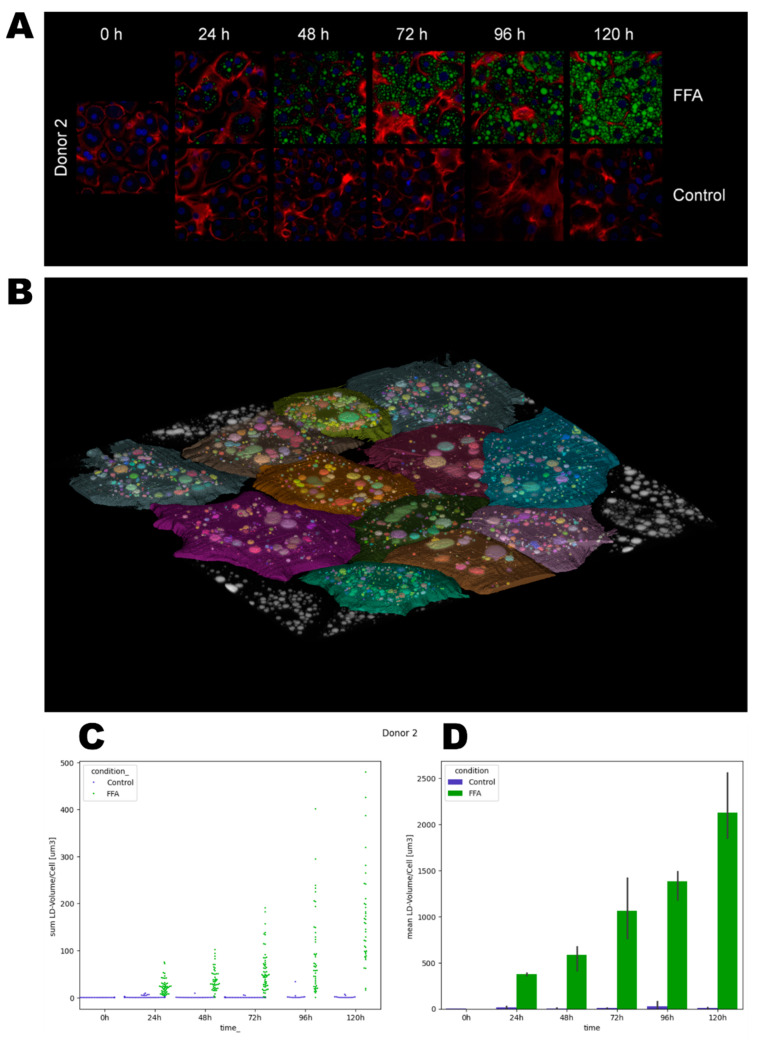
Quantification of lipid droplet growth dynamics. After the cultivation and fixation of cells, LDs were stained with Bodipy (green), cell membranes with Phalloidin iFluor (red), and nuclei with Hoechst (blue). Using a laser scanning microscope, areas were recorded as z-stacks. For bioinformatical analysis, cell borders were determined, and lipid droplet volumes were quantified. (**A**). Representative images of donor 2. (**B**). A representative label map that visualizes the assignment of LDs to individual cells. (**C**). Lipid droplet volumes (µm^3^) per cell. Each data point represents one individual cell. (**D**). Mean lipid droplet volumes (µm^3^) ± SD per condition.

Lipid accumulation was investigated on a cellular level using the fluorescent staining of lipid droplets with the fluorescent dye Bodipy. In addition, we stained cell nuclei with Hoechst and cell boarders/actin filaments with phalloidin. For donor 4, fluorescent microscopy images were only available for time points 0, 24, and 48 h. The high resolution of the fluorescence microscopy allowed the investigation of the dynamics of LD formation (Figure 3A and Supplement Figure S5.

Phalloidin and Hoechst staining allowed the time- and FFA-treatment-dependent investigation of the cell morphology. In general, the borders between neighboring cells were well distinguishable by phalloidin-stained cell membranes. Over time, the cells flattened and formed cell–cell contacts, which can be observed as larger areas between the cells. In addition, in donor 3, we also observed increased actin staining within the hepatocytes. The latter correlates with cellular membrane damage shown by LDH measurements (Supplement Figure S3). Further, we observed clearly visible nuclei, which were often diploid or polyploid, as can be seen by the intensity of the blue fluorescence.

In the second step, the dynamics of LD volumes over time were quantified using imaging and bioinformatical analysis (Figure 3, Supplement Figure S6, and Supplement Table S4). We observed differences in the initial lipid load, such as a higher count of lipid droplets in donor 3 and 4 at 0 h. In the control groups, we observed a loss of the initially present lipid droplets over time. In the FFA-treated cells, the LD volume generally increased over the cultivation period, but notable heterogeneity existed among individual hepatocytes. Notably, cells with both small and large droplets often displayed a spatial organization with smaller droplets near the cell membrane and larger ones closer to the ER (Figure 3A and Supplement Figure S5). Qualitative analysis of the images showed that the cells from donor 1 tended to form either large droplets or a high number of very small droplets. Quantitative analysis revealed that the majority of cells contained a rather small LD volume until 96 h. However, after 120 h, cells exhibited a broader distribution of LD volumes. In contrast, donors 2 and 3 exhibited a more balanced distribution in qualitative analyses, with cells forming both small and large droplets. Quantitatively, we observed a broad distribution of LD volumes across all time points. Cells from donor 4 predominantly formed larger droplets.

Observing the variation in LD sizes and volumes over time, we saw a tendency of cells to first form small-size droplets that seemed to merge in the second step. Afterward, the remaining space in the cytosol was filled with new small LD, as we can see, e.g., in donor 2 from 96 h to 120 h (Figure 3A). In the quantitative data of donor 2, we likewise calculated a smaller standard deviation at 96 h, which increased at 120 h, confirming the qualitative observations (Figure 3D).

### 3.4. Expression of UPR Markers

The expression of different ER stress proteins was determined by Western blotting. As described above, UPR starts with the activation of ATF 6α, IRE 1α, and PERK and leads to autophagy (phosphorylation of JNK, MAP LC3β), fatty acid oxidation (PPARα), inhibition of translation (PERK), or apoptosis (phosphorylation of JNK, CHOP, PARP).

**Figure 4:**
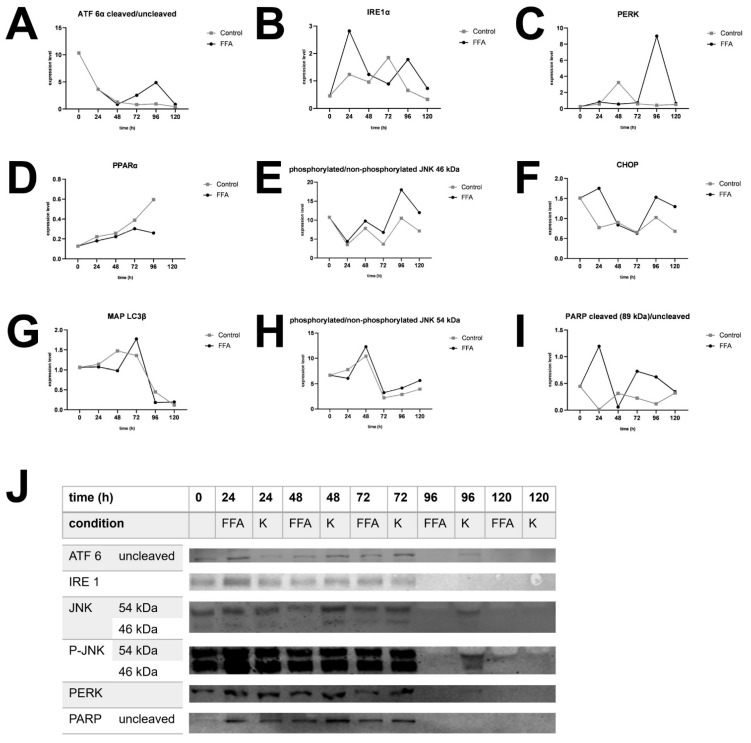
Western blot analysis. Expression of selected UPR markers over 120 h in FFA-treated and control group of different donors. (**A**). Expression of cleaved per uncleaved ATF 6α in donor 1. (**B**). Expression of IRE 1α in donor 4. (**C**). Expression of PERK in donor 2. (**D**). Expression of PPARα in donor 3. (**E**). Expression of phosphorylated per unphosphorylated JNK, isoform sized 46 kDa in donor 4. (**F**). Expression of CHOP in donor 1. (**G**). Expression of MAP LC3β in donor 3. (**H**). Expression of phosphorylated per unphosphorylated JNK, isoform sized 54 kDa in donor 4. (**I**) Expression of cleaved per uncleaved PARP in donor 1. (**J**). Target protein expression of donor 3. After 96 h expression decreased in FFA-treated cells and after 120 h also in control cells.

In general, the investigation of ER-stress markers in FFA-treated and control PHHs showed highly variable expression and activation levels (Figure 4A–I, Supplement Figures S7–S14). In addition, we could observe an initial activation of some of our ER-stress markers at the 0 h time point, e.g., cleavage of ATF 6α, cleavage of PARP, and expression of CHOP (Figure 4A,F,I). The association of these markers with ER stress and apoptosis indicates initial cell stress, which is in line with the observed LDH release (Supplement Figure S3) resulting from post-isolation stress. Similar courses in the activation of PPARα in FFA-treated and control cultures over the whole cultivation time of 120 h indicate a metabolic adaption due to cell culture conditions.

The treatment of PHH cultures with FFA led to an increase in the expression of the ER-stress proteins ATF 6α, IRE 1α, and PERK, as well as related apoptotic downstream targets, e.g., CHOP, PARP, and JNK. However, the activation of these targets varies widely in amplitude and time points in our investigated donors. In contrast, we did not observe a clear FFA-treatment-dependent increase in the expression of MAP LC3β or PPARα in all donors, indicating that regenerative pathways play a lesser role in our in vitro steatosis model.

Donor 3, which was already slightly steatotic at baseline, exhibited a distinct activation pattern. In this donor, UPR activation decreased after 96 h in the FFA-treated group for all markers, although some downstream processes remained active, as seen by the presence of cleavage products. However, the control group also lost its UPR after 120 h (Figure 4J). This suggests that prolonged cultivation conditions may contribute to the observed effect. However, the earlier onset of UPR loss in FFA-treated PHH of donor 3 indicates that an increasing lipid load accelerates this process.

### 3.5. Activation of UPR and Apoptosis Regulates Cell Death Under Severe Steatotic Conditions

**Figure 5:**
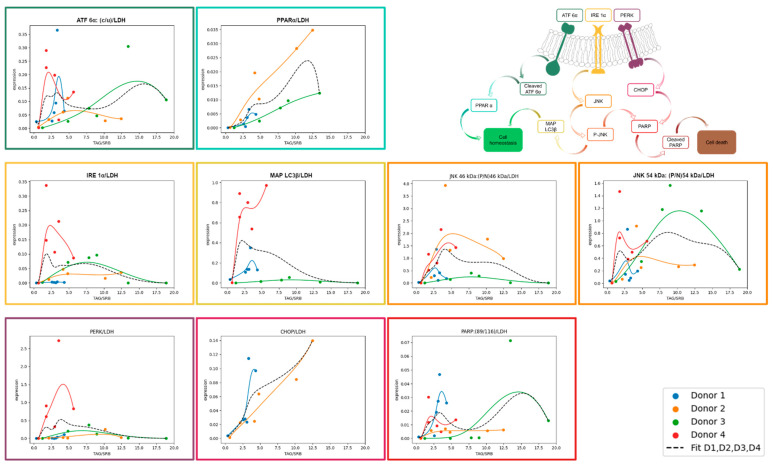
Expression of UPR markers normalized to LDH in relation to TAG/SRB. The expression of selected UPR markers was analyzed with Western blotting in FFA-treated PHH and normalized to total protein staining and positive control. In order to divide UPR signal from other cell stress (e.g., isolation-associated stress), values were divided by LDH expression. Expression values of FFA-treated cells (*y* axis) are shown depending on TAG-content (*x* axis). For trend fitting, we used spline interpolation with Bezier curve.

Similar activation patterns of ER-stress markers in FFA-treated PHH from different donors that vary in their onset and expression suggest a dependency on lipid accumulation. In contrast, we observed similar activation patterns of apoptotic markers related to post-isolation stress. Therefore, we standardized our ER stress markers to the degree of lipid accumulation and adjusted them to account for post-isolation stress. The courses were interpolated using Bezier curve fitting (Figure 5).

#### 3.5.1. ATF 6α-Pathway Activation

After dissociation from BiP, ATF 6α is cleaved inside the Golgi apparatus, producing a cleavage product that enters the nucleus to activate PPARα. In our experiments, ATF 6α activation was lipid-load-dependent but decreased at high TAG content. PPARα is a transcription factor that increases beta oxidation and consequently lowers hepatic lipid load. When comparing ATF 6α and PPARα activation relative to lipid accumulation, we observed that their activation patterns were aligned. However, the extent of ATF 6α activation inversely related with the level of PPARα activation, with the strongest PPARα activation in donor 2. It is known that PPARα is activated by FFA, which explains its increasing activation with increasing cellular lipid content independent from ATF 6α. The further observed increase in PPARα expression in the control PHH shows that the basal culture conditions also induce PPARα activation.

#### 3.5.2. IRE 1α-Pathway Activation

IRE 1α is activated during UPR by dissociation from BiP and subsequently activates JNK via several intermediate steps. We observed a lipid-load-dependent course of IRE 1α activation in donors 2, 3, and 4. In donors 2 and 3, activation remained relatively flat, also with increasing cellular lipid accumulation. In contrast, donor 4 showed a steep increase in IRE 1α activation at low lipid levels. Similar to ATF 6α, IRE 1α activation decreased at higher lipid levels, except in donor 2. These results suggest that IRE 1α activation is donor-dependent in response to increasing lipid load. Downstream of IRE 1α, JNK is phosphorylated and P-JNK can influence apoptosis and downstream autophagy through initiating expression. JNK exists in two isoforms, 46 kDa and 54 kDa, due to differences in splicing. All donors showed JNK activation with a donor-specific maximum at a varying lipid load. The activation patterns of IRE 1α and JNK 54 kDa were in general similar, except for donor 1, displaying JNK 54 kDa activation despite limited IRE 1α activation. In contrast, JNK 46kDa tended to be activated at higher lipid loads than the 54 kDa splicing variant, suggesting a sequential activation in this pathway. Notably, donor 3 exhibited a stronger activation of JNK 54 kDa isoform compared to the 46 kDa isoform. MAP LC3β followed the activation pattern of JNK 54 kDa more closely than that of JNK 46 kDa with the exception of donor 3.

#### 3.5.3. PERK-Pathway Activation

PERK is activated by dissociation from BiP. It leads to a downregulation of mRNA translation which reduces protein load in the already stressed ER. In our analysis, the activation pattern of PERK follows a similar course to that of IRE 1α across all donors. However, the expression level is rather low in donors 1, 2, and 3, also with increasing cellular lipid amount. In contrast donor 4 shows a steep increase in its expression already at a low lipid load which is comparable to the activation patterns of most other investigated UPR proteins. In comparison to ATF 6α and IRE 1α, which show activation already at lower lipid loads, PERK activation takes place when lipid accumulation is more advanced. Prolonged activation of PERK leads to downstream activation of CHOP, a key protein involved in the initiation of apoptosis. CHOP showed a clear increase in expression in hepatocytes from donors 1 and 2 as lipid content rose.

#### 3.5.4. PARP Activation

PARP is part of the common downstream pathway of JNK and CHOP to induce apoptosis. Upon activation, PARP is cleaved into several products, with the 89 kDa cleavage product being the best documented. However, other cleavage products of approximately 40 kDa and 24 kDa have also been described. The 89 kDa PARP fragment was weakly expressed in all of our donors but showed an activation pattern similar to that of JNK. The highest PARP activation was observed in donor 3, which correlates with the observed lipotoxicity. In donor 1, PARP exhibits a decreasing trend at higher lipid levels, a pattern also seen in CHOP. In donor 2, PARP activation stabilizes at a plateau with increasing lipid load. This is similar to the JNK activation pattern, while CHOP increases with rising lipid levels. These results suggest that lipid accumulation, up to a moderate level, leads to strong activation of CHOP but does not necessarily result in a corresponding high activation of PARP 89 kDa.

### 3.6. Increasing Lipid Accumulation Inhibits Cell Proliferation

Our previous results demonstrate that lipid accumulation induces ER stress, accompanied by the activation of the ATF 6α, IRE 1α, and PERK pathways. The activation of PERK at higher lipid loads suggests an inhibition of global protein synthesis, as confirmed by SRB assay for donors 2 and 3 (Supplement Figure S2). On this basis, we hypothesize that lipid accumulation impacts regenerative capacities, specifically cell proliferation.

**Figure 6:**
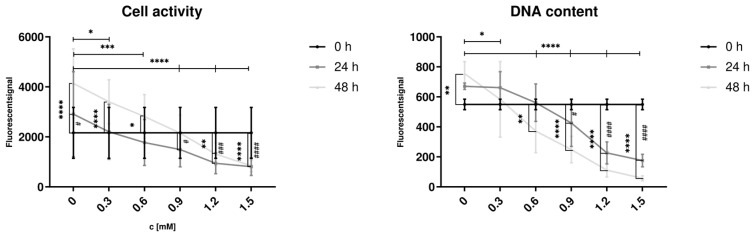
Influence of FFA treatment on cell activity and dsDNA content in proliferating hepatocytes. Hep G2 cells were cultured with varying concentrations of FFA over 48 h, and the medium was changed daily. Alamar Blue Assay (A) was used to investigate cell activity, Sybr Green Assay (B) for dsDNA content. Data are shown as means ± SD, n = 3 biological replicates (including 10 technical replicates per each). Two-way ANOVA and post hoc Tukey’s test was used for statistical analysis. Vertical markers depict significant changes over time: 0 vs. 24 h (####: *p* < 0.0001, ###: *p* < 0.001, #: *p* < 0.05) or 0 vs. 48 h (****: *p* < 0.0001, ***: *p* < 0.001, **: *p* < 0.01, *: *p* < 0.05). Horizontal markers depict significant changes after treatment with varying FFA concentrations in comparison to control (0 mM) after 48 h (****: *p* < 0.0001, ***: *p* < 0.001, **: *p* < 0.01, *: *p* < 0.05).

To investigate hepatic cell proliferation, we used HepG2 cells as model for proliferating hepatocytes, as mature primary hepatocytes do not exhibit noteworthy proliferation in vitro. Cell proliferation in HepG2 cells was assessed using the Alamar Blue assay (Figure 6A), which measures cell activity, and Sybr Green assay (Figure 6B), which detects changes in DNA content by binding to dsDNA as a surrogate for cell count.

Hep G2 cells were treated with varying concentrations of FFA (0 to 1.5 mM) to induce steatosis. In the control group (0 mM FFA), a significant increase in cell activity and dsDNA content was observed after 48 h of culture, indicating an increased cell count. Low FFA concentrations (0.3 and 0.6 mM) were associated with increased cell activity; however, dsDNA content showed no significant change at 0.3 mM. Treatment with FFA concentrations of 0.6 mM or higher resulted in a decrease of dsDNA content after 48 h, with cell activity significantly reduced at 1.2 and 1.5 mM after both 24 and 48 h.

After 48 h, significant differences in cell activity and dsDNA content were observed between the control group (0 mM) and all FFA-treated groups. These findings indicate that even low concentrations of FFA reduce cell proliferation, while higher concentrations exert cytotoxic effects on proliferative hepatocytes.

## 4. Discussion

The regenerative capacity of the liver following resection is impaired by obesity and high-saturated-fat diets, as demonstrated in animal models [4,5]. Similarly, clinical observations indicate that overweight and obese patients exhibit increased postoperative rates of acute liver failure, complications, and mortality [3,34,35,36]. As demonstrated in our previous study, ER stress is one of the most relevant consequences of hepatic lipid accumulation and is more prominently activated than oxidative stress [9]. The aim of the present study was to further investigate the effects of prolonged lipid accumulation on lipid metabolism, as well as the downstream impact of ER stress on regeneration and cell death [11,13].

### 4.1. Initial Lipid Load Influences the Dynamics of Further Lipid Accumulation

In this study, we investigated PHH isolated from resected liver tissues from four patients. FFA-treated PHH cultures from all donors exhibited significant lipid accumulation compared to both the control group and their initial lipid load. Consistently, the highest lipid accumulation occurred during the first 24 h, aligning with findings from our previous study [9]. Notably, donor 1, who had the lowest initial lipid load, showed the greatest relative increase in lipid accumulation during this period. However, in terms of absolute values, lipid accumulation was dependent on the initial load, with a higher starting lipid content still resulting in greater lipid accumulation during steatosis induction. Additionally, the quantitative imaging analyses confirmed that the capability of individual hepatocytes to accumulate lipids is distributed heterogeneously. Single cells store lipids more rapidly and earlier during the cultivation period, while others maintain lower lipid levels over several days. This pattern, where initially leaner cells exhibit a higher relative lipid accumulation while fatty cells maintain higher absolute lipid levels, has also been observed in previous studies [9,37]. Overall, the lipid accumulation patterns observed in our four donors align with established mechanisms.

### 4.2. FFA Uptake Was Independent of Existing Lipid Load and Cultivated PHH Used a Small Part of FFAs to Form TAG

MASLD is caused by lipid accumulation inside hepatocytes, which is influenced by lipid offer, intake, metabolization, storage and VLDL excretion. Cells incorporated a substantial portion of the available FFA, with similar uptake observed across all donors. The uptake was the highest during the first 24 h of cultivation but decreased over time. In vivo, FFA uptake is partially mediated by specialized transmembrane transporters such as CD36 and specific isoforms of free acid transport proteins (FATPs). Recent studies have shown increased expression of these transporters in MASLD or in hepatic cell lines incubated with oleic or palmitic acid [38,39]. While the variation in initial lipid load among our donors suggests potential differences in transporter-dependent FFA uptake, the uniform uptake observed in our study indicates that FFA uptake in vitro may occur independently of these transporters. Consistent with our findings, a study investigating the velocity of fatty acid uptake in hepatic cells from obese and control rats found no significant differences between these groups [40]. Insulin is a known extracellular factor influencing FFA uptake, but recent studies suggest the involvement of further extracellular factors [41]. Our results indicate that intracellular factors may also play a role. Although FFA uptake was similar across donors, the patterns of lipid accumulation and TAG excretion varied. Donors 3 and 4 exhibited lower TAG storage rates, while donors 1 and 2 converted a larger portion of FFA into intracellular TAG. The metabolization of FFA to TAG is dependent on intracellular activation of FFA to acyl-Co A and three esterifications on glyceraldehyde 3-phosphate (G3P). The first esterification is speed-determining for TAG synthesis and dependent on G3P acyltransferase enzymes. However, there are still several pathways that channel off metabolites to the construction of other lipids, e.g., phosphatidylethanolamine, lecithin, or cardiolipins [42]. Our results suggest that donors 3 and 4 are either less able to activate FFA or channel off more lipid metabolites to formation of other lipids. Regarding TAG excretion, donors 1, 2, and 4 showed higher excretion levels in the FFA-treated group compared to controls, whereas donor 3 exhibited the opposite, with higher TAG excretion in the control group. These findings confirm previous results from Fon Tacer and Rozman, who reported increased VLDL secretion under MASLD conditions but the inhibition of VLDL secretion under MASH conditions [43]. Recent studies suggest that these differences depend on ER function, as VLDL formation in hepatocytes relies on the transfer of triglycerides to ApoB100 in the ER lumen [38]. In rodent studies, moderate ER stress led to increased ApoB100 secretion, whereas excessive ER stress decreased it [44].

Furthermore, only a portion of FFA was utilized for TAG formation with the gap between FFA uptake and TAG synthesis increasing over time. In a previous study, we demonstrated higher expression of lipid metabolism proteins, such as ACSL1 and CPT1L, in steatotic hepatocytes, indicating enhanced FFA metabolism under steatotic conditions [45]. However, we also observed that basal metabolic rates in hepatocytes remained relatively constant but decreased under prolonged steatotic conditions [9]. We hypothesize that FFA could be partly transformed into phospholipids. Clinical data from adipose patients show an increased risk of bile stone formation due to an imbalance in bile composition [46,47,48]. Phospholipids were an essential part of bile, and the excretion of excess FFA as phospholipids via bile could be a pathological effect of disturbed lipid homeostasis.

### 4.3. Lipid Accumulation Activates UPR and Especially the Expression of Apoptosis-Associated Markers

Increasing lipid accumulation is associated with the induction of ER stress, which initiates the UPR. Throughout all donors, we observed an initial activation of several UPR markers simultaneously with high initial LDH activity in the supernatant. A high LDH activity after cell isolation was also observed in other studies as a result of post-isolation stress [9,49]. As a consequence, we normalized our UPR expression data to LDH.

Over time, we observed an activation of various UPR markers. Our results suggest a predominance of apoptosis-inducing pathways over repair mechanisms within the UPR. Visualizing this as a scale, the two opposing sides represent repair and apoptosis. On the repair side of the scale, the ATF 6α/PPARα pathway emerged as a key player. Both ATF 6α and PPARα showed a lipid-load-dependent course. However, we also observed a time-dependent increase in PPARα expression in the control cells, suggesting that PPARα activation can occur naturally, even in the absence of additional FFA treatment. This likely reflects a basal, low-level activation of PPARα under normal metabolic conditions, possibly related to inherent lipid content. On the opposing side of the scale, which tilts toward apoptosis and cell death, lies the PERK/CHOP/PARP pathway. PERK and CHOP expressions were clearly associated with higher lipid loads, indicating that the apoptotic cascade is activated upstream. However, PARP activation, a clear marker of apoptosis execution, did not follow this pattern consistently across all donors. PARP was markedly activated only in donor 3, where its expression coincided with signs of lipotoxicity and cell death, as evidenced by LDH release and microscopy analyses. In contrast, in donors 1, 2, and 4, PARP activation remained limited, suggesting that these cells were on the brink of apoptosis or experiencing only slight activation of apoptotic pathways. JNK, a downstream marker of IRE 1α activation, serves as a pivot weight on this scale, determining the balance between autophagy and apoptosis [17]. Although we did not observe strong activation of the autophagy marker MAP LC3β, which could tip the scale toward repair, we saw a parallel activation of the apoptosis-associated PARP in donor 3, further shifting the balance decisively toward cell death. While autophagy is known to suppress apoptosis under certain conditions [50], repair mechanisms appeared inactive in our model. The observed activation of JNK and CHOP highlights the progressive tipping of the scale toward lipoapoptosis, a process resulting from prolonged ER stress. Similar results were shown in other studies using cellular human and animal models [51,52,53]. In rat hepatocytes, palmitate led to a higher expression of CHOP and increased cell death. This study also suggests that there must be additional pathways inducing cell death independent of CHOP [53]. Another study observed an activation of caspase-dependent apoptosis after JNK-phosphorylation in primary mouse hepatocytes [51]. An in vivo model of high-fat-diet-fed rats found an increase in ER-stress, CHOP, and caspase activation [5].

Given the use of primary human hepatocytes, donor-specific characteristics must be considered. Donor 1 was the youngest, and donor 4 was the oldest, with both suffering from benign liver diseases. Donors 2 and 3, by contrast, were operated on for malignant liver tumors. All donors except donor 2 were normal in weight and female. Notably, donor 3, despite having the lowest BMI, exhibited the highest initial lipid load, suggesting individual susceptibility to lipid accumulation. Donors 3 and 4 showed distinct characteristics. Donor 4 displayed elevated UPR marker expression even at low lipid loads, which may align with findings in mice showing age-related increases in ER stress [53]. For donor 3, UPR marker expression declined in FFA-treated cells after 96 h, a trend that became apparent in control cells at 120 h. As we normalized target protein expression to total intracellular protein, this decrease was not attributable to overall protein degradation but was specific to ER stress proteins. Concurrently, microscopy revealed a reduction in cell count and cell surface proteins over time, with these effects occurring earlier in FFA-treated cells compared to controls. Importantly, the decline in UPR markers preceded an increase in LDH release, suggesting that donor 3 reached a stage of lipotoxicity characterized by membrane damage. This membrane disruption could explain the observed LDH release and may also facilitate the release of TAG, which, under such conditions, might not be packaged and excreted as VLDL.

Together, these findings suggest that donor 3 represents a critical case where severe lipid accumulation leads to UPR downregulation, loss of cellular integrity, and the transition from stress to apoptosis. This reinforces the earlier conclusion that apoptosis execution occurs primarily under conditions of extreme lipid load, where the balance decisively tilts toward cell death. The pharmacological targeting of UPR pathways to shift the balance from apoptosis to repair could therefore contribute to control of the progression from MASLD to MASH. So far, therapeutic options concentrate on lowering the cellular lipid load. For example, fibrates targeting PPARα have been in use for hyperlipidemia therapy for years now. By stimulating PPARα, they increase fatty acid uptake, the conversion to acyl-CoA derivatives, and catabolism by mitochondrial fatty acid oxidation [54]. Additional attempts tried to address apoptosis by inhibiting caspase but were not effective in in vivo studies [55]. The pathophysiological role of autophagy in liver function is emerging as a novel therapeutical target in metabolic disease conditions but so far not fully understood. The selective targeting of the signaling proteins that lie at the tipping point between autophagy and apoptosis, like JNK, could thus be the basis for future autophagy-based therapies [56].

### 4.4. Cell Growth and Cell Activity Are Disturbed by FFA Treatment

UPR shows high interindividual variability, influenced by factors such as initial lipid load, dynamics of lipid intake, metabolization, and excretion. If ER stress fails to restore cellular homeostasis, MASLD can progress to MASH and other complications [52]. Moreover, MASLD is associated with impaired liver regeneration following liver resection [5].

Our results demonstrated that lipid accumulation strongly activates ER stress. Prolonged ER stress, driven by sustained PERK activation, leads to a global inhibition of protein translation, impairing both cellular repair and tissue regeneration. This challenge is particularly critical in the context of severe lipid accumulation, which triggers cell death and underscores the need for effective recovery mechanisms to preserve liver integrity under lipotoxic conditions.

To further investigate the effects of lipid accumulation on hepatic cell proliferation, we used HepG2 cells as a model. The Alamar Blue and Sybr Green assays revealed that low FFA concentrations (0.3 and 0.6 mM) initially increased cell activity, likely reflecting metabolic adaptation to moderate lipid exposure. However, DNA content at 0.3 mM remained unchanged, indicating that this increase in activity did not correspond to effective proliferation. At 0.6 mM or higher, both DNA content and cell activity declined, with significant reductions observed at 1.2 and 1.5 mM, suggesting a dose-dependent relationship where low FFA concentrations disrupt proliferation and higher concentrations exert cytotoxic effects. We based our in vitro steatosis model on the results presented by Gómez-Lechón et al., who systematically analyzed lipotoxic effects of different ratios and concentrations of oleate and palmitate-exposure on PHH and HepG2 cells [25]. With regard to their results, we used a 2:1 ratio of oleate and palmitate and remained below 2 mM. Here, we did not investigate cytotoxic effects but rather proliferation. Considering our results, the tipping point between adaptation and negative effects on proliferation is situated around 0.6 mM of the applied FFA mixture.

Extrapolated to the organ level, these findings suggest that the lipid-induced disruption of cell proliferation and activity could compromise liver regeneration and, in severe cases, lead to organ failure. This is consistent with clinical observations of increased postoperative complications and mortality in patients with progressive steatosis undergoing liver resection [3]. Notably, cell proliferation appears more sensitive to steatosis than cell activity. At low FFA concentrations (0.3–0.6 mM), cell activity increased over time, likely reflecting metabolic adaptation but DNA content either remained stable (0.3 mM) or declined (0.6 mM). This indicates that cells under mild lipid stress may prioritize metabolic functions over division. At higher FFA concentrations (≥1.2 mM), both cell activity and proliferation were significantly reduced, consistent with previous reports of diminished metabolic activity under FFA treatment [57]. Further studies in rat hepatocytes confirmed our observations, showing suppressed cell proliferation and increased cell death under treatment with saturated fatty acids [58]. These effects were paralleled by a CHOP activation, similar to our findings in primary hepatocytes.

Interestingly, the observed increase in cell activity in low FFA-treated groups between 24 and 48 h may represent a transient adaptive response before metabolic stress overwhelms repair mechanisms. Together, these findings underscore the detrimental impact of lipid accumulation on cellular proliferation and liver regeneration, highlighting the critical need to address lipid-induced stress in the treatment of steatosis-related liver diseases.

### 4.5. Limitations

Our study is limited by the use of primary human hepatocytes (PHH) and the small number of donors included in the experiments. The use of PHH inherently introduces variability due to differences in donor age, sex, underlying diseases, lifestyle, and medication. In particular, our results highlight the influence of initial lipid content on cellular responses, which required us to account for the donor-specific lipid load in our analysis. Despite these challenges, the consistency of our findings across different experimental read-outs supports the validity of our conclusions.

The limited availability of primary hepatic cells also constrained the number of parallel experiments, making it difficult to perform extensive subgroup analyses. Additionally, inter-donor variability could not be entirely controlled, emphasizing the need for further studies using larger donor pools to validate these findings. Furthermore, prolonged cultivation times up to 120 h are known to induce dedifferentiation processes and cultivation stress in PHH [23], as seen in the slight activation of UPR targets also in the control cells.

In addition to PHH, we used HepG2 cells as a model to investigate lipid-induced stress and proliferation. While HepG2 cells are suitable for studying steatosis due to their ability to increase lipid load in response to fatty acid treatment, they differ from non-cancerous hepatocytes in key aspects. HepG2 cells respond differently to glucose and fructose treatment compared to primary hepatocytes [37], and their capacity for very low-density lipoprotein (VLDL) secretion is notably poor [57]. These differences may limit the translatability of some findings to physiological or in vivo conditions. Nonetheless, HepG2 cells provide a valuable tool for investigating lipid-induced cellular stress and remain a widely used model for such studies.

## 5. Conclusions

This study underscores the detrimental impact of lipid accumulation on hepatocyte function, highlighting its role in activating ER stress pathways, inhibiting regeneration, and inducing apoptosis. Prolonged ER stress, driven by sustained PERK activation, disrupts cellular repair and tissue regeneration, particularly under severe lipid load. These findings align with clinical observations of impaired liver regeneration and increased postoperative complications in obese patients undergoing liver resection.

Additionally, the gap between FFA uptake and TAG synthesis suggests the diversion of FFA into alternative pathways, such as phospholipid synthesis. This may contribute to imbalances in bile composition, offering a potential explanation for the increased risk of gallstone formation in patients with disturbed lipid metabolism.

In future studies, we will further investigate the metabolic fate of unstored FFA and the kinetics of LD dynamics. In addition, we aim to transfer our data into a mathematical model that allows a comprehensive display of lipid homeostasis and the simulation of the impact of lipid accumulation on cell fate. These strategies will improve our understanding of lipid-induced hepatocyte dysfunction, which will be crucial for developing targeted therapies to enhance liver regeneration and mitigate complications in steatosis-related liver diseases.

## Figures and Tables

**Table 1:** Donor characteristics.

Donor	Age	Sex	Diagnosis	BMI (kg/m^2^)
Donor 1	39	Female	FNH ^1^	19.9
Donor 2	68	Male	PHC ^2^	30
Donor 3	71	Female	iCCA ^3^	18
Donor 4	85	Female	XGC and BH ^4^	19.2

^1^ Focal nodular hyperplasia; ^2^ Perihilar cholangiocarcinoma; ^3^ Intrahepatic cholangiocarcinoma; ^4^ XGC: Xanthogranulomatous cholecystitis; BH: Biliary hamartoma.

## Data Availability

The datasets generated during and/or analyzed during the current study are available from the corresponding author on reasonable request.

## Supplementary Materials

The following supporting information can be downloaded at https://www.mdpi.com/article/10.3390/cells14060449/s1. Table S1: Technical data of fluorescent microscopy and bioinformatic evaluation, Table S2: Technical data for Western blots, Table S3: FFA-metabolization to TAG, Table S4: Bioinformatic evaluation of lipid droplets, Figure S1: Initial Confluence, Figure S2: Quantification of cell surface protein with SRB, Figure S3: LDH activity, Figure S4: Intracellular TAG, Figure S5: Dynamics of lipid droplet formation, Figure S6: Bioinformatic analysis of lipid droplet growth dynamics, Figure S7: PARP expression, Figure S8: CHOP expression, Figure S9: PERK expression, Figure S10: JNK and P-JNK expression, Figure S11: MAP LC3β expression, Figure S12: IRE 1α expression, Figure S13: PPARα expression, Figure S14: ATF 6α expression
