# Supplementary material for: Cell Homeostasis or Cell Death—The Balancing Act Between Autophagy and Apoptosis Caused by Steatosis-Induced Endoplasmic Reticulum (ER) Stress
Source: Cells. 2025 Mar 18;14(6):449. doi: 10.3390/cells14060449 (PMC11941029; doi:10.3390/cells14060449)
Supplement: Supplementary file 1 [file cells-14-00449-s001.zip › cells-3412236-supplementary.pdf]

Supplement

Tables

Table S1: Technical data of fluorescent microscopy and bioinformatic evaluation

Condition	Count of image stacks acquired	Count of image stacks segmented and segmentation method [3,4]	Condition	Count of image stacks acquired	Count of image stacks segmented and segmentation method [3,4]
Donor 1, 0 h	5	5 [3]	Donor 3, 0 h	5	5 [3]
Donor 1, 24 h, FFA[1]	5	5 [3]	Donor 3, 24 h, FFA[1]	5	3 [3]
Donor 1, 24 h, C[2]	5	5 [3]	Donor 3, 24 h, C[2]	5	3 (2 [3] and 1 [4])
Donor 1, 48 h, FFA	5	5 [3]	Donor 3, 48 h, FFA	5	3 (1 [3] and 2 [4])
Donor 1, 48 h, C	5	5 [3]	Donor 3, 48 h, C	5	3 [4]
Donor 1, 72 h, FFA	5	5 [3]	Donor 3, 72 h, FFA	5	3 (1 [3] and 2 [4])
Donor 1, 72 h, C	5	4 [3]	Donor 3, 72 h, C	5	3 [4]
Donor 1, 96 h, FFA	5	3 (2 [3] and 1 [4])	Donor 3, 96 h, FFA	5	3 (1 [3] and 2 [4])
Donor 1, 96 h, C	5	3 [3]	Donor 3, 96 h, C	5	3 [4]
Donor 1, 120 h, FFA	5	3 (2 [3] and 1 [4])	Donor 3, 120 h, FFA	2	0 (no cells left)
Donor 1, 120 h, C	5	4 [3]	Donor 3, 120 h, C	5	0 (not necessary as no FFA-data)
Donor 2, 0 h	5	4 [3]	Donor 4, 0 h	6	6 [3]
Donor 2, 24 h, FFA[1]	5	3 [3]	Donor 4, 24 h, FFA[1]	5	3 [3]
Donor 2, 24 h, C[2]	5	5 [3]	Donor 4, 24 h, C[2]	5	4 [3]
Donor 2, 48 h, FFA	5	3 (2 [3] and 1 [4])	Donor 4, 48 h, FFA	5	3 [4]
Donor 2, 48 h, C	5	3 (2 [3] and 1 [4])	Donor 4, 48 h, C	5	3 [4]
Donor 2, 72 h, FFA	5	3 [3]			
Donor 2, 72 h, C	5	5 [3]			
Donor 2, 96 h, FFA	5	3 (2 [3] and 1 [4])			
Donor 2, 96 h, C	5	3 (2 [3] and 1 [4])			
Donor 2, 120 h, FFA	5	3 (2 [3] and 1 [4])			
Donor 2, 120 h, C	5	3 [4]			

[1] FFA-treated group, [2] Control group, [3] segmented with Cellpose 2.5D segmentation approach, [4] segmented with 3D slicer

Table S2: Technical data for Western blots

Protein	Antibody product number and manufacturer	Dilution	Running Gel	Protein amount [µg]	Positive control[1]	Secondary Antibody
**IRE 1α** (Inositol-requiring Enzyme 1α)	Cell Signaling Technology (Danvers, USA), 3294	1:1000	12%	20	TUN[2] 5 µg/ml, 24 h	Anti-rabbit
**MAP LC3β** (Microtubule-associated protein-light chain 3)	Santa Cruz Biotechnology (Santa Cruz, USA), I2519	1:500	15%	20	TG[3] 100 nM, 16 h	Anti-mouse
**JNK** (Januskinase)	Cell Signaling Technology (Danvers, USA), 9252	1:1000	15%	25	TG 100 nM, 16 h	Anti-rabbit
**P-JNK** (Phospho-Januskinase)	Cell Signaling Technology	1:1000	15%	20	TG 100 nM, 24 h	Anti-rabbit

	(Danvers, USA), 9251					
**ATF 6α** (Activating transcription factor 6α)	Santa Cruz Biotechnology (Santa Cruz, USA), G1919	1:100	12%	20	TG 2.5 nM, 48 h	Anti-mouse
**PPARα** (Anti-Peroxisome Proliferator Activated Receptor Alpha)	Cusabio (Houston, USA), CSB-PA030137-100	1:1000	12%	17	TG 2.5 nM, 48 h	Anti-rabbit
**PERK** (Protein Kinase RNA-like Endoplasmic Reticulum Kinase)	abcam (Cambridge, UK), ab79483	1:1000	12%	25	TUN 5 µg/ml, 48 h	Anti-rabbit
**CHOP** (C/EBP-homologous protein)	Santa Cruz Biotechnology (Santa Cruz, USA), K2618	1:250	15%	25	TUN 5 µg/ml, 48 h	Anti-mouse
**PARP** (Poly (ADP-ribose) polymerase)	Cell Signaling Technology (Danvers, USA), 9542	1:1000	12%	20	TG 100 nM, 24 h	Anti-rabbit

[1] HepG2 cells cultivated in medium containing a definit concentration of Tunicamycin or Thapsigargin for a period of time, [2] Tunicamycin; [3] Thapsigargin, [4]

Table S3: FFA-metabolization to TAG

Condition	FFA uptake n [µmol] [1]	TAG stored n [µmol] [2]	TAG excretion n [µmol] [3]	Formation of intracellular TAG [µmol] [4]	FFA [µmol] to intracellular TAG [5]	Formation of excreted TAG [µmol] [6]	FFA [µmol] to excreted TAG [7]	Gap of FFA uptake to TAG formation [µmol] [8]
**Donor 1**								
**0 h**		0,126	0					
**24 h, FFA**	1,172	0,534	0,095	0,408	1,224	0,020	0,061	-0,113
**24 h, C**		0,207	0,075					
**48 h, FFA**	2,279	0,591	0,202	0,465	1,395	0,041	0,122	0,763
**48 h, C**		0,081	0,161					
**72 h, FFA**	3,374	0,673	0,331	0,547	1,642	0,084	0,251	1,481
**72 h, C**		0,071	0,248					
**96 h, FFA**	4,398	0,686	0,435	0,560	1,681	0,106	0,319	2,398
**96 h, C**		0,080	0,329					
**120 h, FFA**	5,312	0,857	0,541	0,731	2,194	0,131	0,393	2,725
**120 h, C**		0,074	0,410					
**Donor 2**								
**0 h**		0,114	0					
**24 h, FFA**	1,150	0,261	0,095	0,147	0,442	0,026	0,077	0,632
**24 h, C**		0,051	0,070					
**48 h, FFA**	2,095	0,566	0,180	0,451	1,354	0,030	0,091	0,650
**48 h, C**		0,053	0,150					
**72 h, FFA**	2,902	0,369	0,257	0,255	0,764	0,043	0,128	2,010
**72 h, C**		0,060	0,214					
**96 h, FFA**	3,649	0,999	0,356	0,885	2,656	0,072	0,215	0,778
**96 h, C**		0,075	0,284					
**120 h, FFA**	4,365	0,556	0,435	0,442	1,327	0,070	0,211	2,828
**120 h, C**		0,059	0,365					
**Donor 3**								

**0 h**		0,198	0					
**24 h, FFA**	1,119	0,394	0,095	0,195	0,586	-0,011	-0,032	0,564
**24 h, C**		0,089	0,105					
**48 h, FFA**	2,157	0,527	0,200	0,329	0,987	-0,036	-0,108	1,278
**48 h, C**		0,068	0,236					
**72 h, FFA**	3,011	0,491	0,295	0,293	0,879	-0,040	-0,121	2,254
**72 h, C**		0,089	0,335					
**96 h, FFA**	3,810	0,451	0,394	0,252	0,757	-0,044	-0,131	3,183
**96 h, C**		0,056	0,437					
**120 h, FFA**	4,586	0,305	0,514	0,107	0,321	0,001	0,002	4,263
**120 h, C**		0,102	0,514					
**Donor 4**								
**0 h**		0,145	0					
**24 h, FFA**	1,164	0,224	0,066	0,079	0,237	0,006	0,018	0,909
**24 h, C**		0,125	0,060					
**48 h, FFA**	2,281	0,201	0,141	0,055	0,165	0,013	0,040	2,076
**48 h, C**		0,090	0,127					
**72 h, FFA**	3,317	0,301	0,214	0,155	0,466	0,022	0,067	2,784
**72 h, C**		0,076	0,192					
**96 h, FFA**	4,335	0,268	0,285	0,123	0,368	0,030	0,089	3,879
**96 h, C**		0,062	0,255					
**120 h, FFA**	4,893	0,280	0,355	0,135	0,404	0,039	0,118	4,371
**120 h, C**		0,045	0,316					

Data of FFA-treated (FFA) and control (C) group at different time points. To improve readability values are rounded to three decimal places

[1] FFA uptake was calculated by the difference of offered FFA to remaining FFA in the supernatant and summed up over the time, [2] Stored TAG was measured in cell lysates of FFA- treated and control group; [3] Excreted TAG was measured in cell supernatant of FFA-treated and control group and summed up over the time, [4] Formation of LD was calculated by the difference of stored TAG in FFA-treated group to 0 h-condition, [5] Amount of FFA, that is metabolized to LD, was calculated by three times Formation of LD, [6] Formation of VLDL out of added FFA was calculated by the difference of TAG in the supernatant of FFA-treated and control group, [7] Amount of FFA, that is metabolized to VLDL, was calculated by three times Formation of VLDL, [8] Amount of FFA, that is not metabolized to TAG, was calculated by the difference of FFA uptake to the sum of FFA, that is metabolized to LD and VLDL

Table S4: Bioinformatic evaluation of lipid droplets

**Condition**	**Count of segmented cells**	**Count of cells containing LD**	**LD volume (µm³)**	**Mean LD volume (µm³) per cell[1]**	**Mean LD size (µm³)**	**SD of LD size (µm³)**
**Donor 1**						
0 h	132	90	415,6408	4,618231	83,12815	63,3888
24 h FFA	136	135	24218,3	179,3948	4843,661	576,1791
24 Control	112	61	208,1891	3,412936	41,63782	38,00457
48 h FFA	120	120	56381,49	469,8458	11276,3	3214,155
48 h Control	134	92	3118,474	33,89646	623,6949	463,3624
72 h FFA	128	128	46483,93	363,1557	9296,785	4122,015
72 h Control	61	29	619,0834	21,34771	154,7709	185,8802
96 h FFA	61	61	31435,84	515,3417	10478,61	6028,939
96 h Control	63	37	1068,137	28,86856	356,0455	510,7922
120 h FFA	40	40	74568,51	1864,213	24856,17	12654,76

Condition	Count of segmented cells	Count of cells containing LD	LD volume (µm³)	Mean LD volume (µm³) per cell[1]	Mean LD size (µm³)	SD of LD size (µm³)
120 h Control	100	52	927,6378	17,83919	231,9094	240,3937
**Donor 2**						
0 h	69	48	98,75601	2,057417	24,689	21,59088
24 h FFA	47	47	17773,14	378,152	5924,381	1004,877
24 Control	81	47	1148,947	24,44569	229,7895	276,591
48 h FFA	45	44	26733,92	607,5891	8911,307	4515,85
48 h Control	54	27	165,5308	6,130769	55,17692	36,37688
72 h FFA	54	54	55717,48	1031,805	18572,49	3575,331
72 h Control	110	51	414,2472	8,122493	82,84943	69,01785
96 h FFA	44	44	57090,24	1297,505	19030,08	11390,14
96 h Control	27	14	351,5533	25,11095	117,1844	167,8433
120 h FFA	38	38	78757,31	2072,561	26252,44	4925,673
120 h Control	25	16	156,991	9,811935	52,33032	69,31655
**Donor 3**						
0 h	92	87	2733,965	31,42488	546,793	321,5633
24 h FFA	70	69	21085,54	305,5876	7028,514	5332,506
24 Control	35	34	3293,998	96,88229	1097,999	261,2661
48 h FFA	39	38	42789,09	1126,029	14263,03	5587,511
48 h Control	22	14	914,8015	65,34297	304,9338	258,356
72 h FFA	35	35	23808,51	680,2432	7936,171	1810,875
72 h Control	13	11	154,9037	14,08215	77,45183	11,7766
96 h FFA	46	46	55914,51	1215,533	18638,17	2478,398
96 h Control	13	11	177,2171	16,11065	88,60856	75,52712
**Donor 4**						
0 h	72	68	5554,375	81,68198	925,7291	703,6441
24 h FFA	40	39	37271,75	955,686	12423,92	2126,264
24 Control	56	51	3812,085	74,74676	953,0212	337,0013
48 h FFA	22	22	29543,37	1342,881	9847,791	5527,339

Condition	Count of segmented cells	Count of cells containing LD	LD volume (µm³)	Mean LD volume (µm³) per cell[1]	Mean LD size (µm³)	SD of LD size (µm³)
48 h Control	20	12	679,2526	56,60438	226,4175	94,87995

[1]Calculated by division of "count of LD" by "count of cells containing LD"

Figures

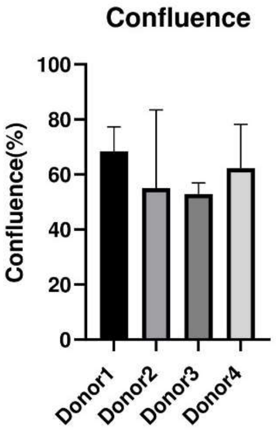

Figure S1: Initial Confluence

PHH were seeded and attached overnight. Initial confluence was measured in three pictures per donor. Data are shown as means +SD, n=3. One-way ANOVA and post hoc Tukey's test was used for statistical analysis. There was no significant difference between the donors.

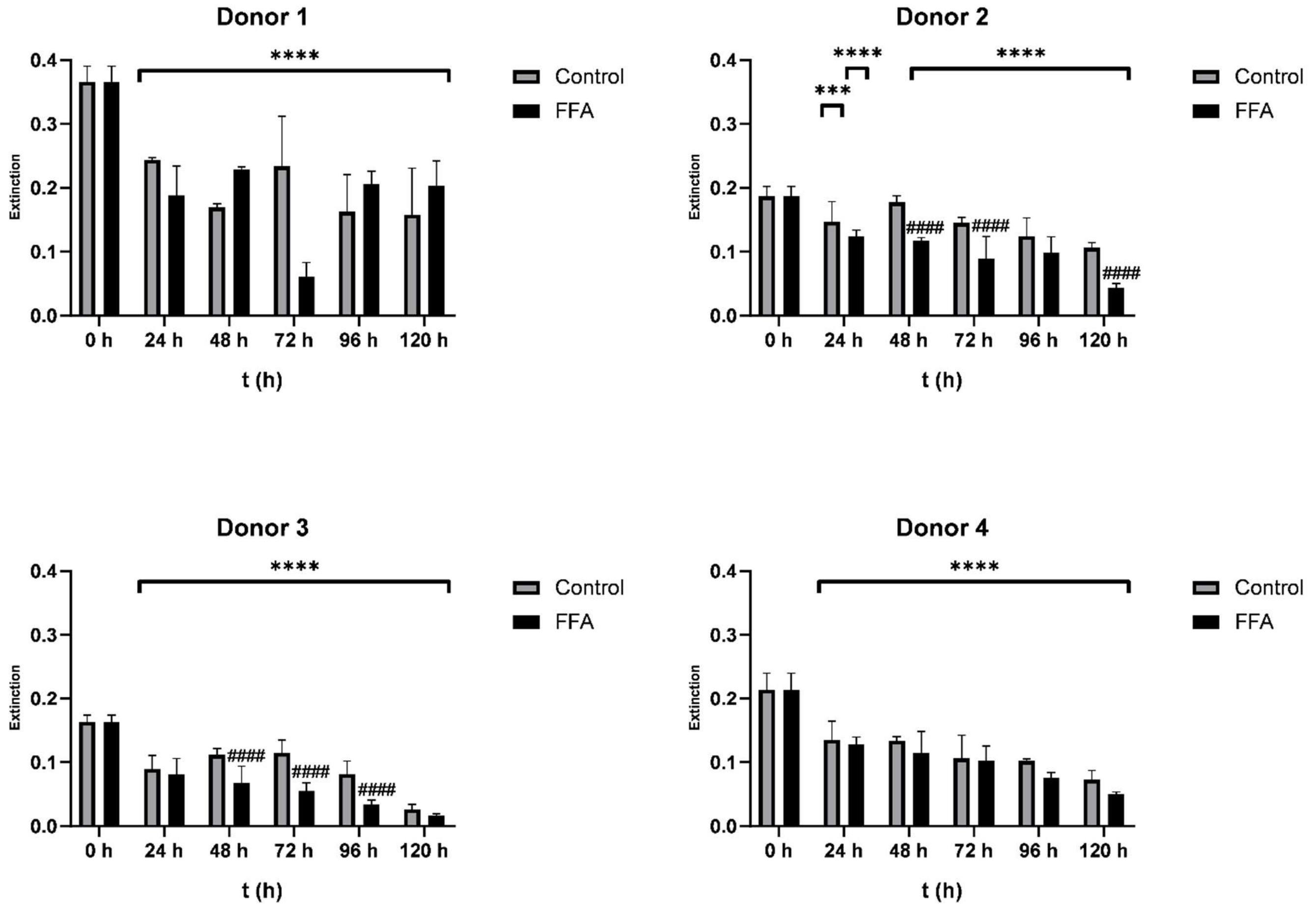

Figure S2: Quantification of cell surface protein with SRB

PHH were cultivated under treatment of FFA containing or control medium. After certain periods of time cells were fixed. For semiquantitative analysis of cell surface protein they were stained with SRB and extinction was measured to determine relative concentration. ROUT test at Q=1% was used to identify outliers. Data are shown as means +SD, n=8 (Donor 1) or n=12 (Donor 2, 3 and 4) technical replicates. Two-way ANOVA and post hoc Tukey's test was used for statistical analysis. Differences between the start and other time points were analyzed (****: $p<0.0001$, ***: $p<0.001$) as well as differences between FFA-treated and control group (####: $p<0.0001$). In donor 1 we observed a cell decrease in the well of 72 h-point in FFA-treated group, this value must be seen as faulty.

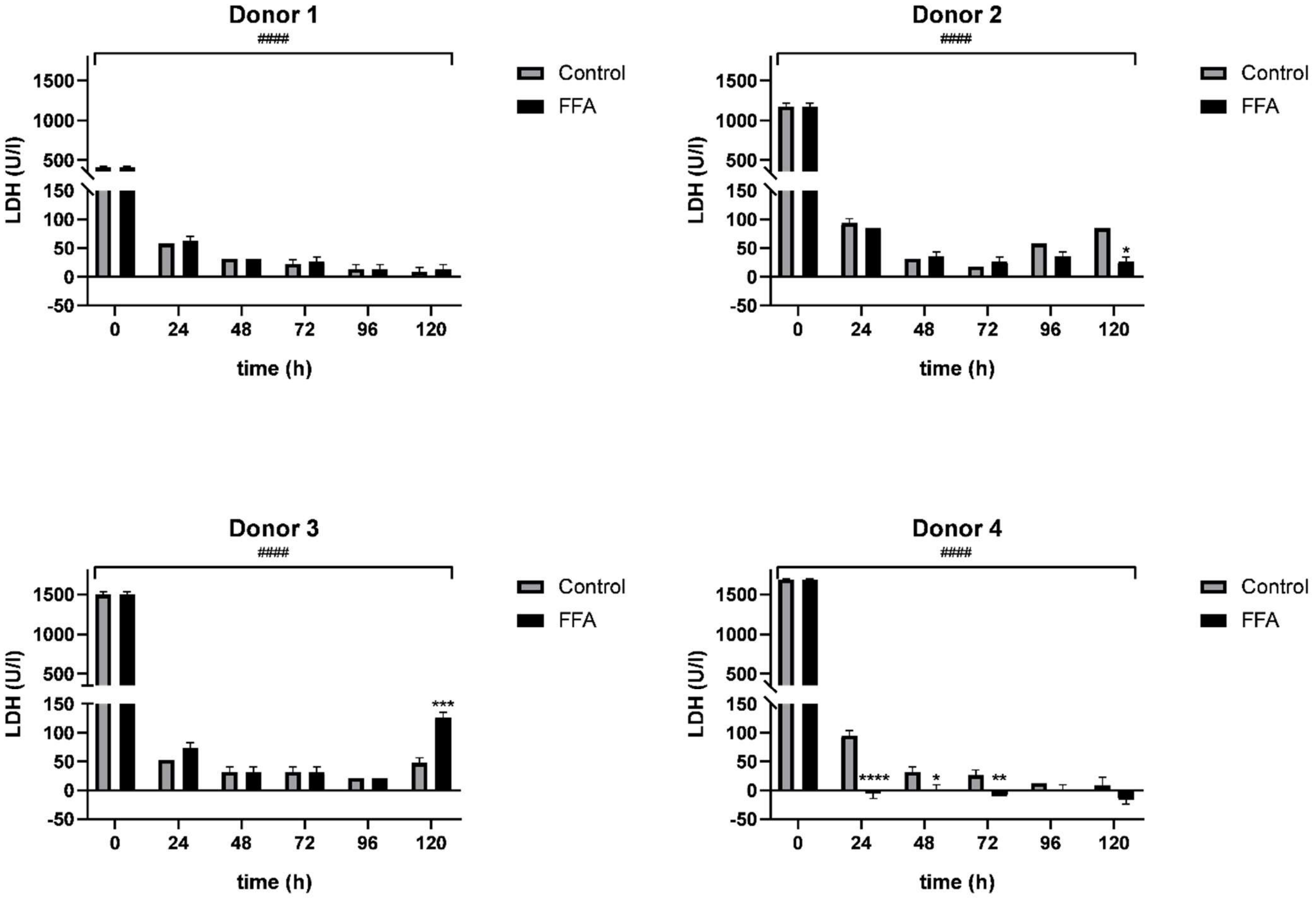

Figure S3: LDH activity

Lactate dehydrogenase (LDH) is an intracellular enzyme usually located in the cytoplasm of hepatocytes and other cell types. In cases of membrane damage which occur e.g. based on cell necrosis LDH is released. Activity of LDH was measured in supernatants of FFA-treated and control group PHH. Data are shown as means +SD, n=3 technical replicates. Two-way ANOVA and post hoc Tukey's test was used for statistical analysis. In all donors we could observe a significant higher excretion of LDH during the first 24 h (####: $p<0.0001$). There were very few significant differences between FFA-treated and control group (****: $p<0.0001$, ***: $p<0.001$, **: $p<0.01$, *: $p<0.03$)

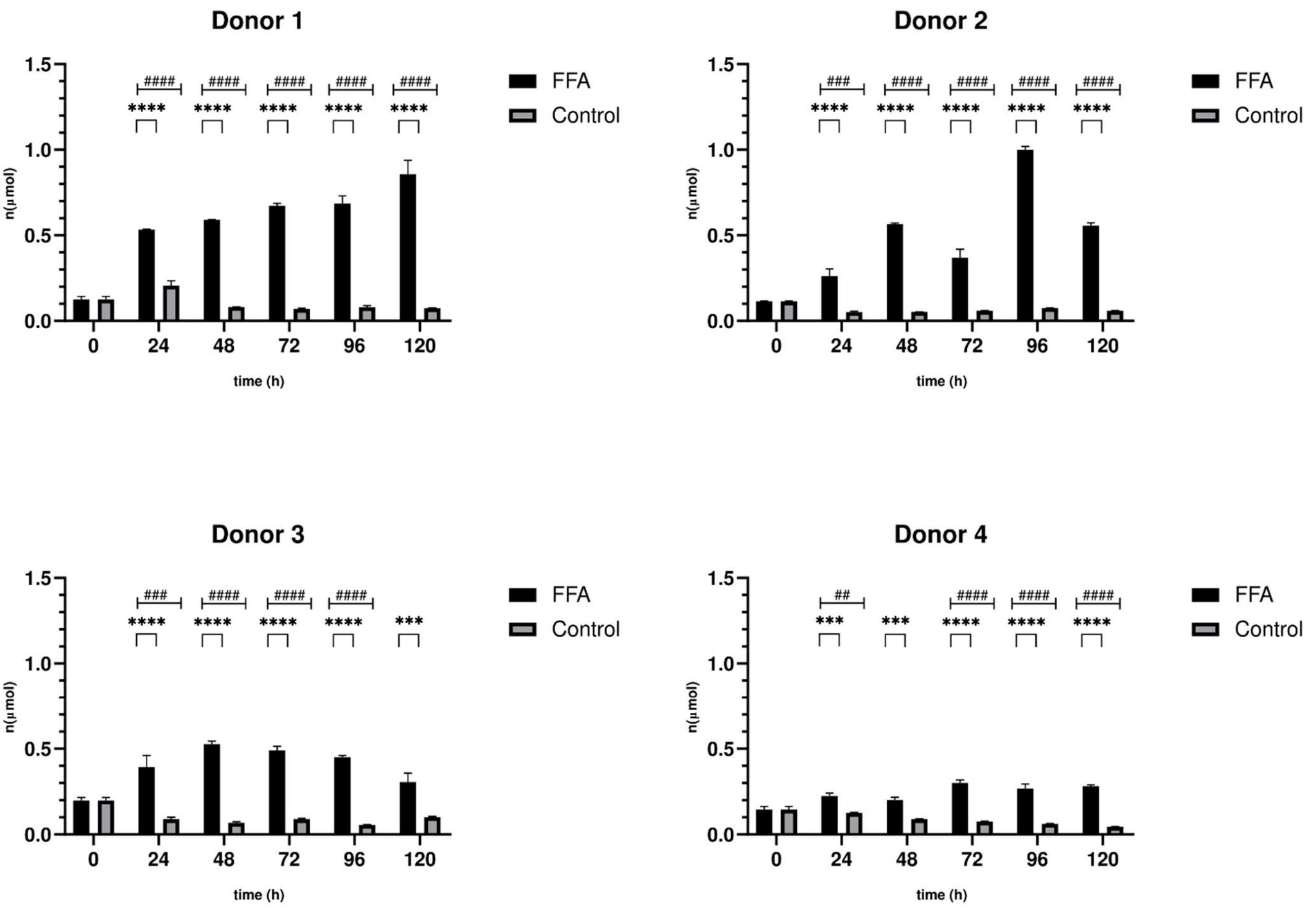

Figure S4: Intracellular TAG

Intracellular TAG was measured in cell lysate of PHH of FFA-treated and control group at different point of time. Data are shown as means +SD, n=2 technical replicates. Two-way ANOVA and post hoc Tukey's test was used for statistical analysis. FFA-treated groups show a significant increase compared to control at the same time points (####: p<0.0001, ###: p<0.001, ##: p<0.01) and a significant increase related to the start (****: p<0.0001, ***: p<0.001) at almost all points of time.

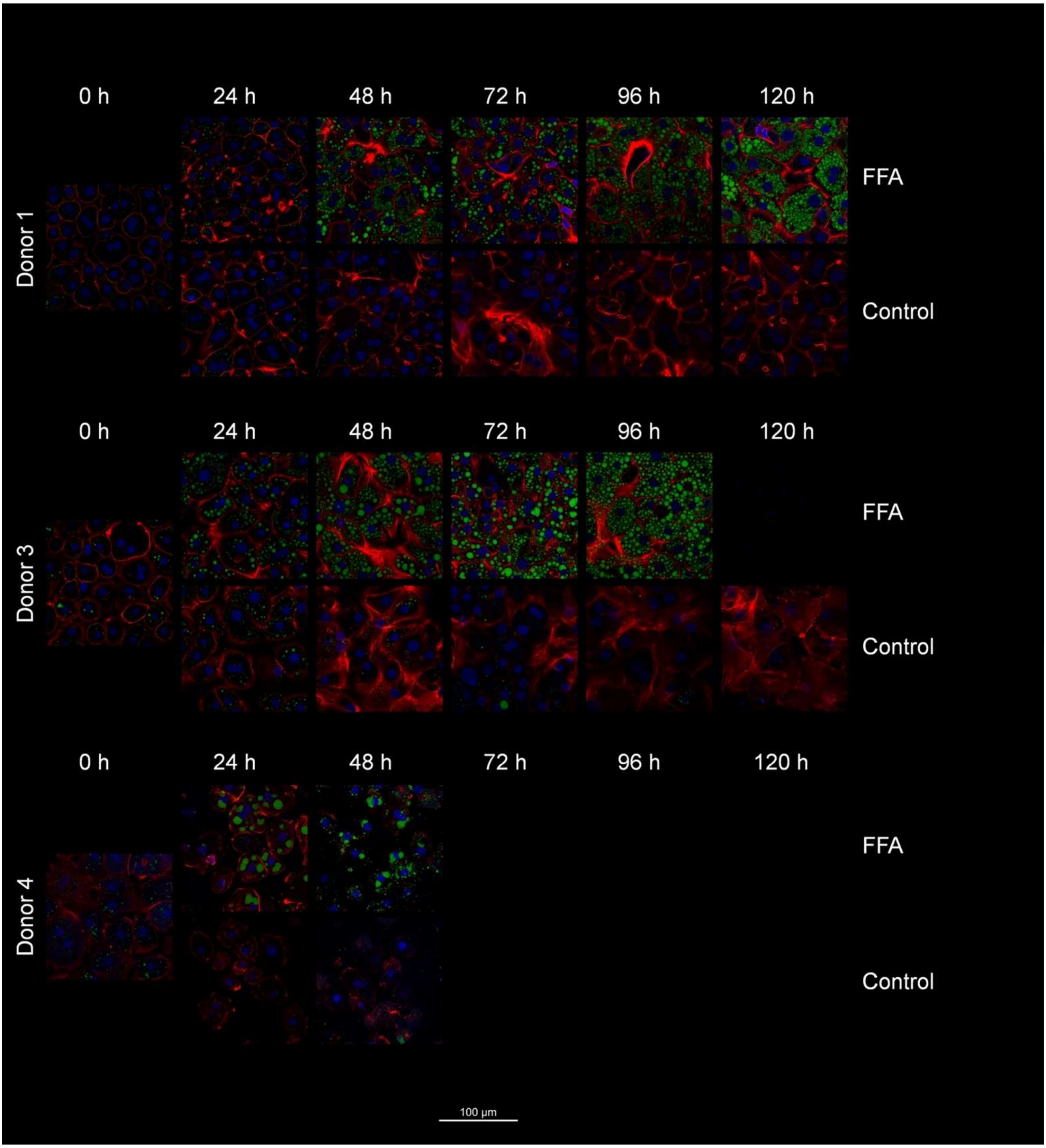

Figure S5: Dynamics of lipid droplet formation
After cultivation and fixation of cells, LD were stained with Bodipy (green), cell membrane with Phalloidin iFluor (red) and nuclei with Hoechst (blue). Using a laser scanning microscope, n=5 areas per condition were recorded as z-stacks. One representative picture per condition was selected for this figure.

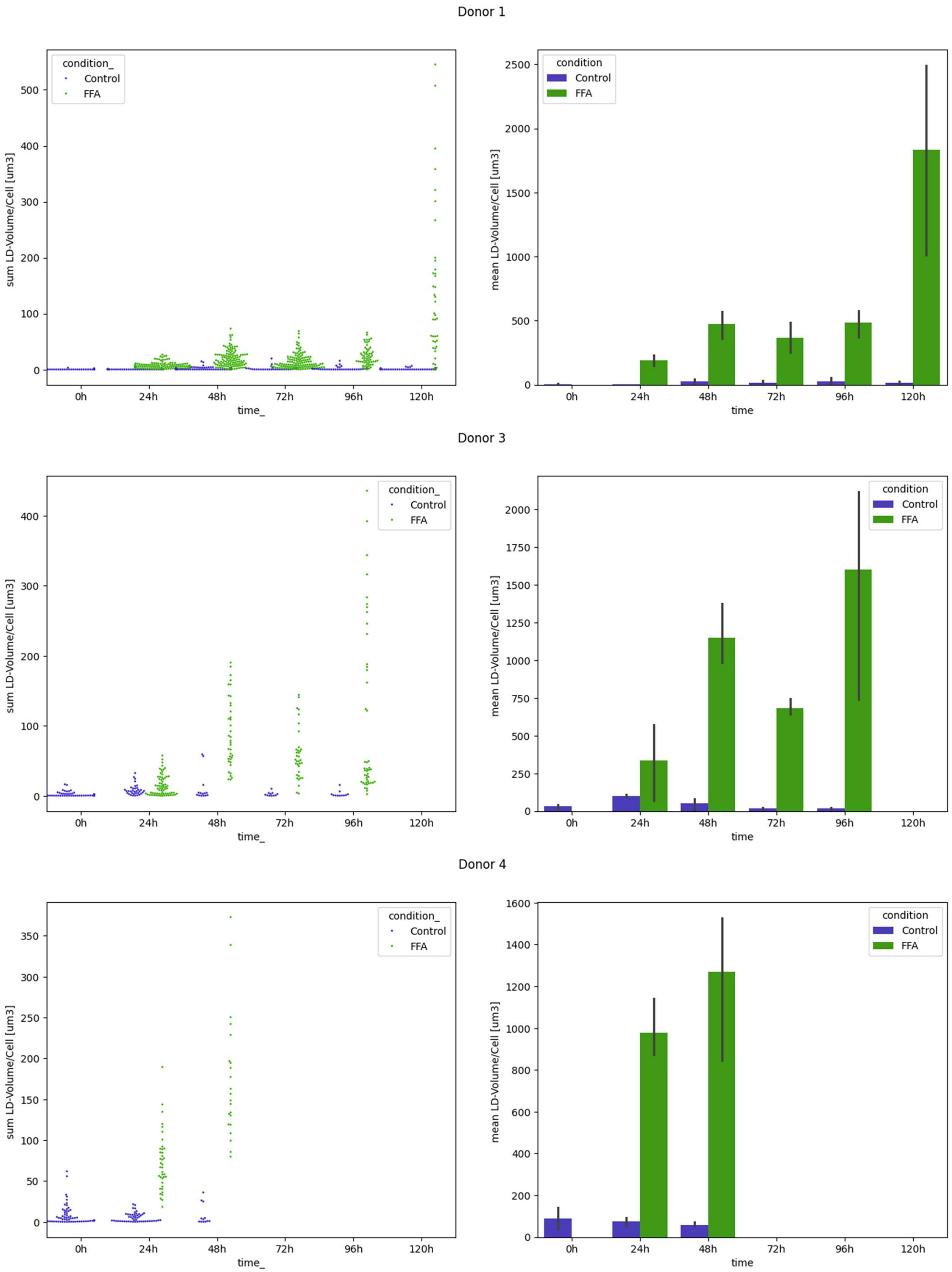

Figure S6: Bioinformatic analysis of lipid droplet growth dynamics

For bioinformatical analysis cell borders were determined and lipid droplet volumes were quantified. The first column shows lipid droplet volumes (μm³) per cell. Each data point represents one individual cell. The second column shows mean lipid droplet volumes (μm³) ± SD per condition.

Western Blots

PARP

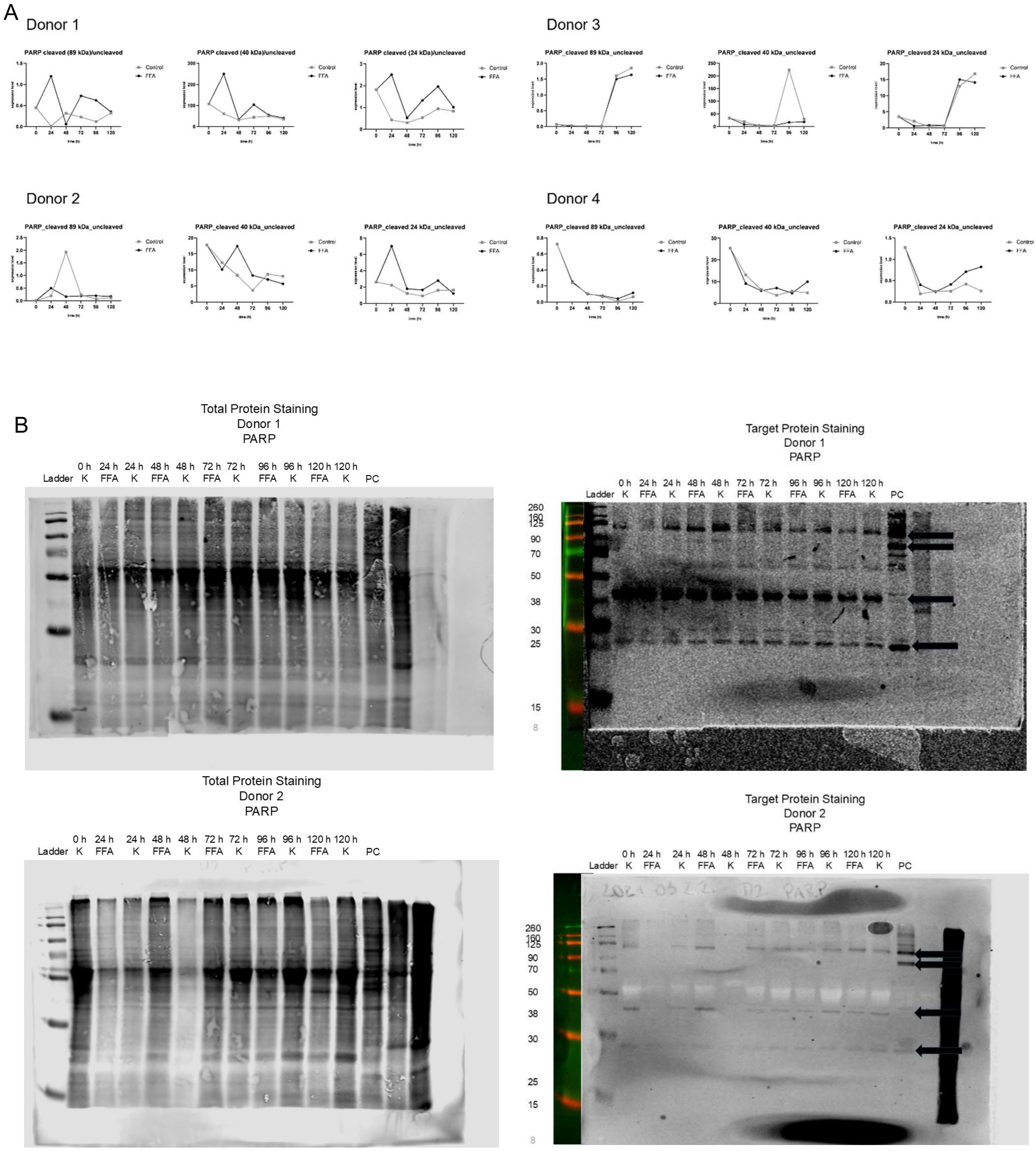

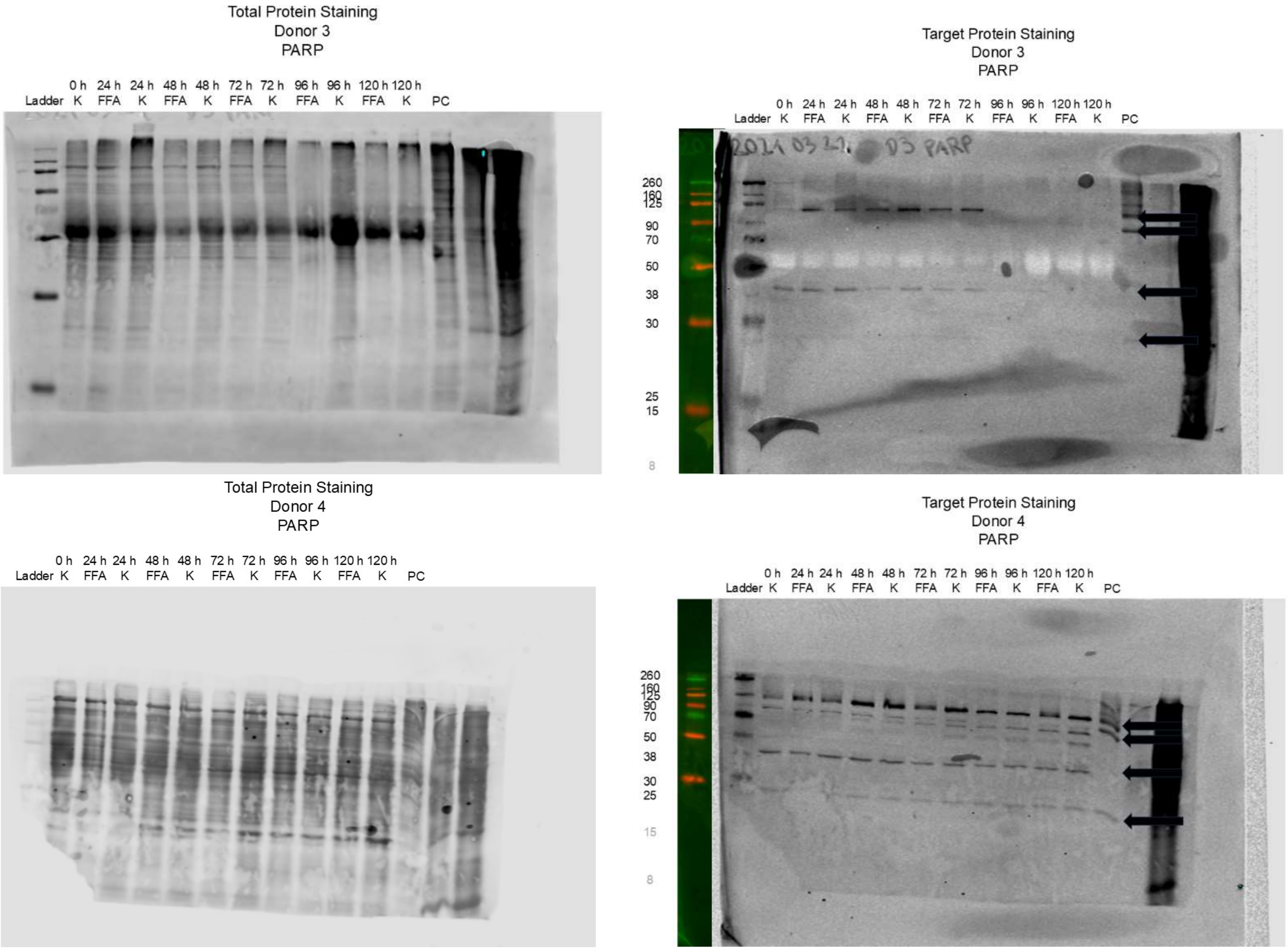

Figure S7: PARP expression

A. Expression of cleaved (89 kDa, 40 kDa and 24 kDa) per uncleaved PARP (116 kDa) in FFA-treated and control group

B. Total and target protein staining of each donor

CHOP

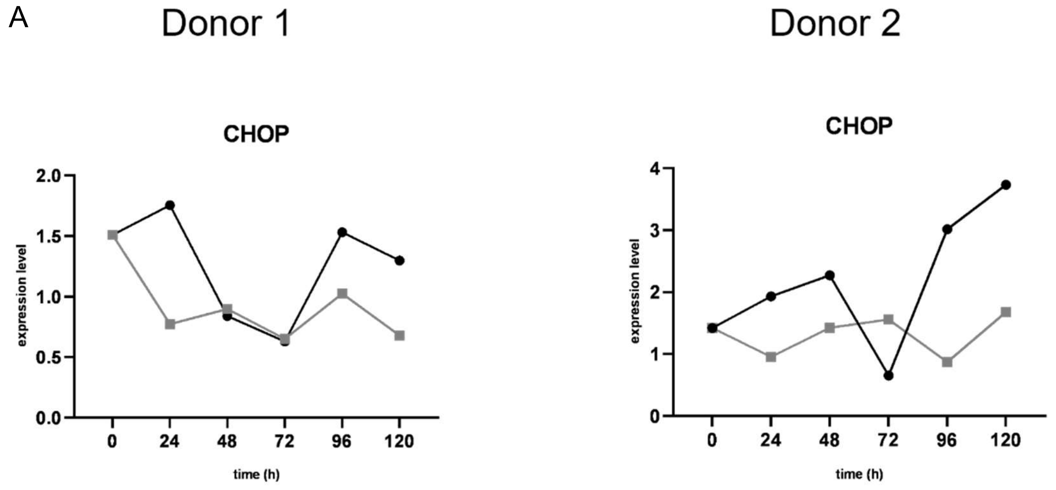

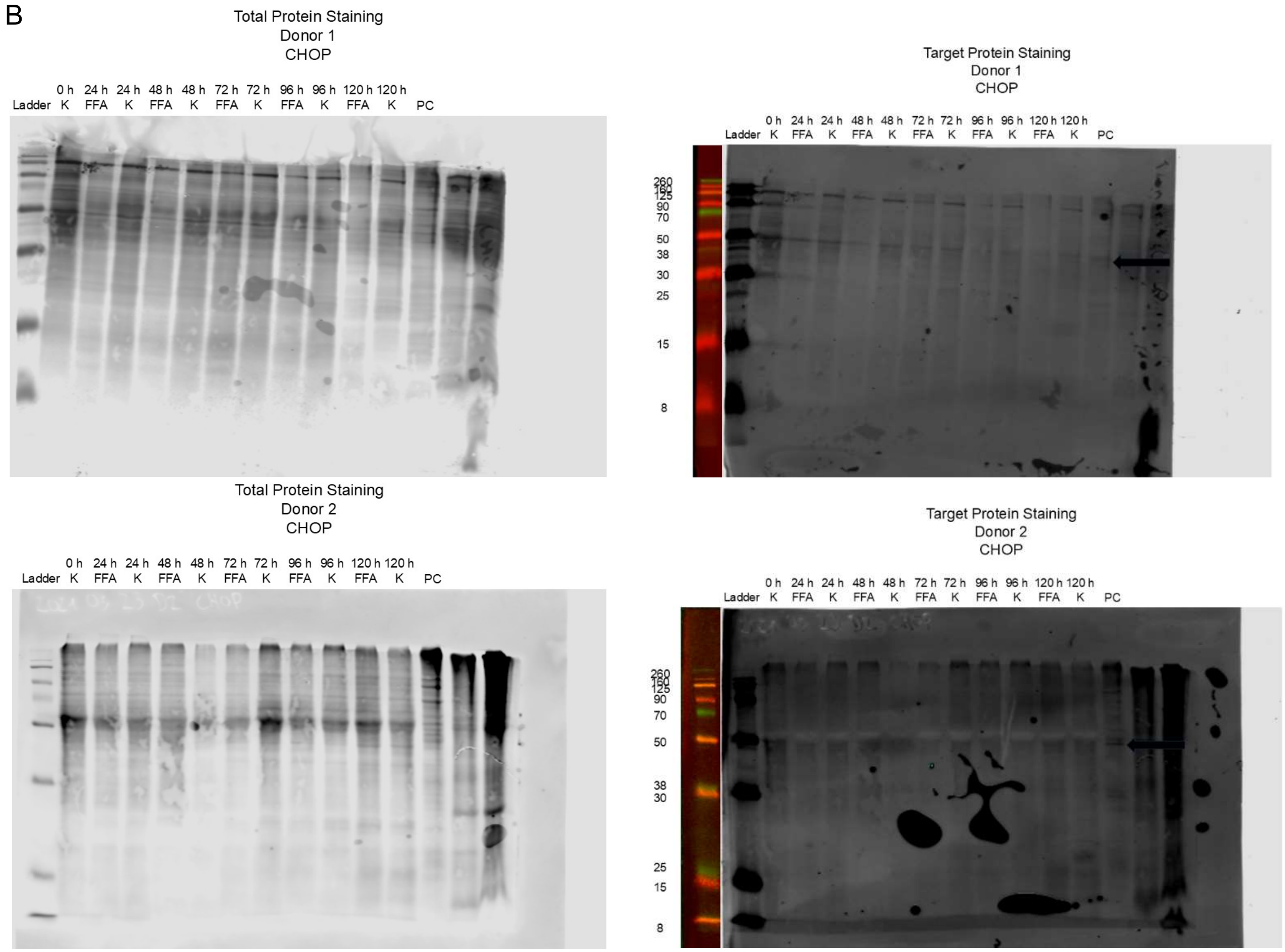

Figure S8: CHOP expression

A. Expression of CHOP in FFA-treated and control group

B. Total and target protein staining

PERK

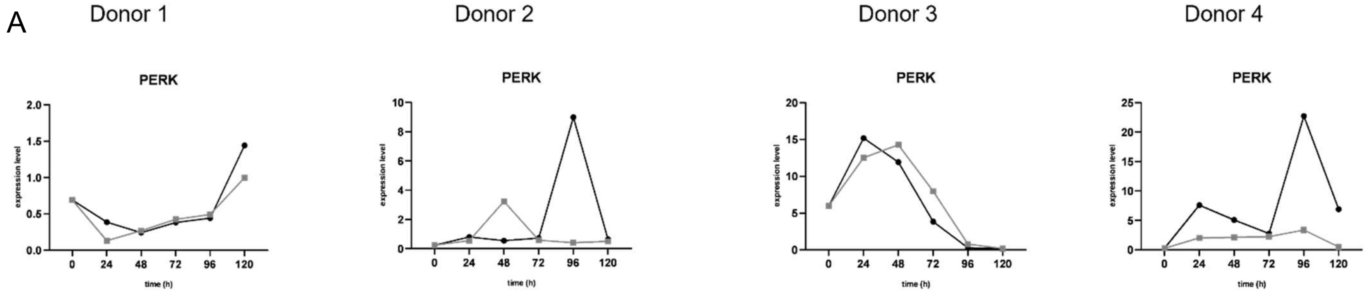

B

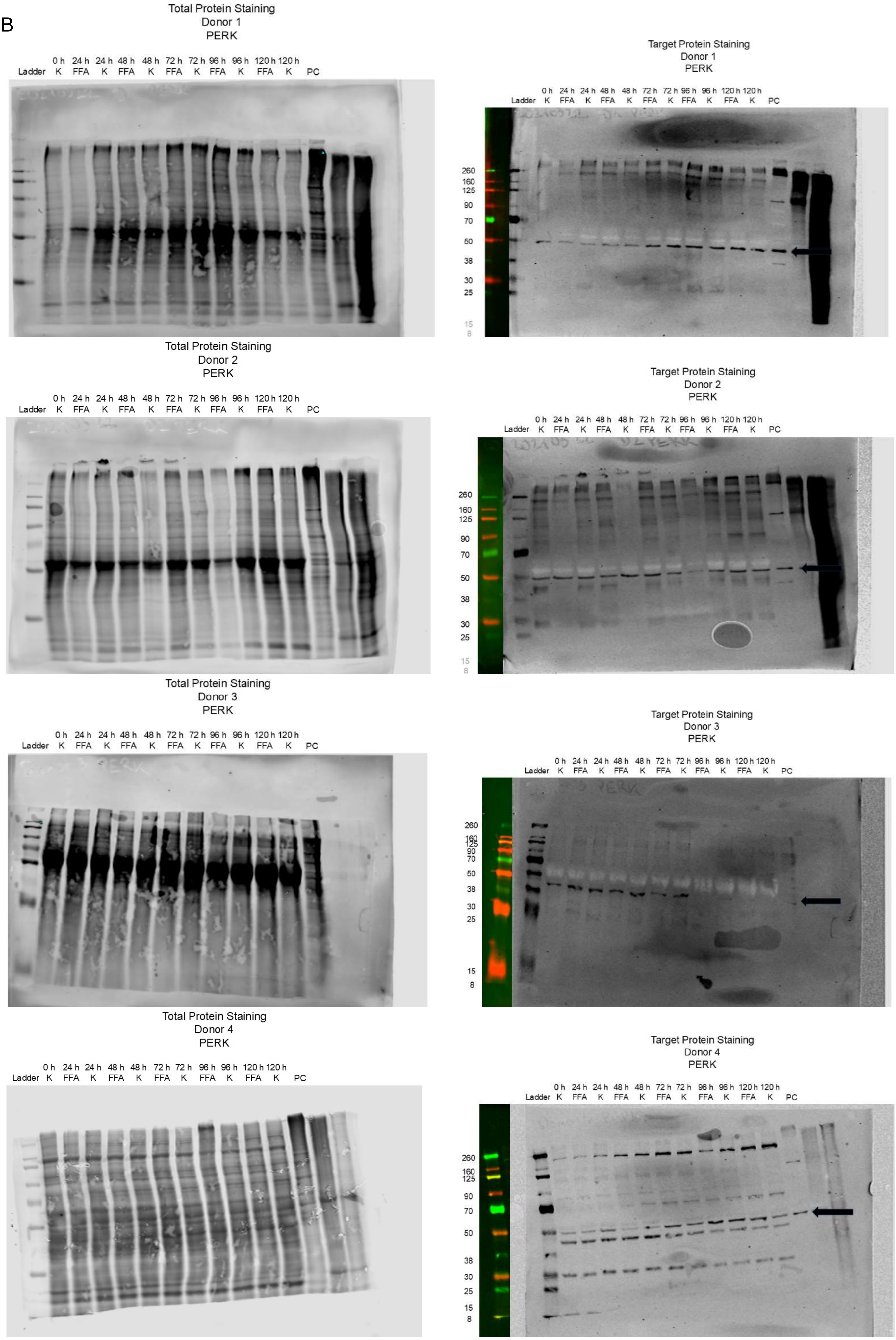

Total Protein Staining
Donor 1
PERK
Ladder 0 h K 24 h FFA 24 h K 48 h FFA 48 h K 72 h FFA 72 h K 96 h FFA 96 h K 120 h FFA 120 h K PC
Target Protein Staining
Donor 1
PERK
Ladder 0 h K 24 h FFA 24 h K 48 h FFA 48 h K 72 h FFA 72 h K 96 h FFA 96 h K 120 h FFA 120 h K PC
260
160
125
90
70
50
38
30
25
15
8
Total Protein Staining
Donor 2
PERK
Ladder 0 h K 24 h FFA 24 h K 48 h FFA 48 h K 72 h FFA 72 h K 96 h FFA 96 h K 120 h FFA 120 h K PC
Target Protein Staining
Donor 2
PERK
Ladder 0 h K 24 h FFA 24 h K 48 h FFA 48 h K 72 h FFA 72 h K 96 h FFA 96 h K 120 h FFA 120 h K PC
260
160
125
90
70
50
38
30
25
15
8
Total Protein Staining
Donor 3
PERK
Ladder 0 h K 24 h FFA 24 h K 48 h FFA 48 h K 72 h FFA 72 h K 96 h FFA 96 h K 120 h FFA 120 h K PC
Target Protein Staining
Donor 3
PERK
Ladder 0 h K 24 h FFA 24 h K 48 h FFA 48 h K 72 h FFA 72 h K 96 h FFA 96 h K 120 h FFA 120 h K PC
260
160
125
90
70
50
38
30
25
15
8
Total Protein Staining
Donor 4
PERK
Ladder 0 h K 24 h FFA 24 h K 48 h FFA 48 h K 72 h FFA 72 h K 96 h FFA 96 h K 120 h FFA 120 h K PC
Target Protein Staining
Donor 4
PERK
Ladder 0 h K 24 h FFA 24 h K 48 h FFA 48 h K 72 h FFA 72 h K 96 h FFA 96 h K 120 h FFA 120 h K PC
260
160
125
90
70
50
38
30
25
15
8

Figure S9: PERK expression

A. Expression of PERK in FFA-treated and control group

B. Total and target protein staining of each donor

JNK and P-JNK

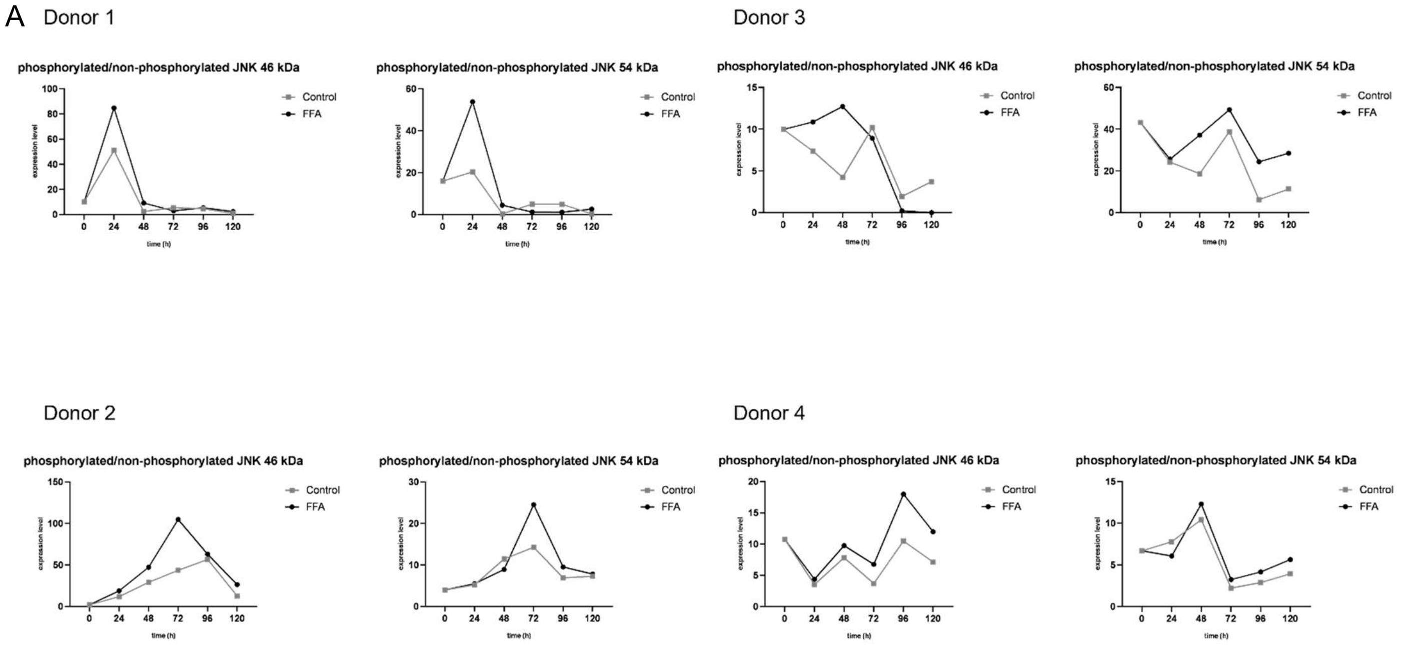

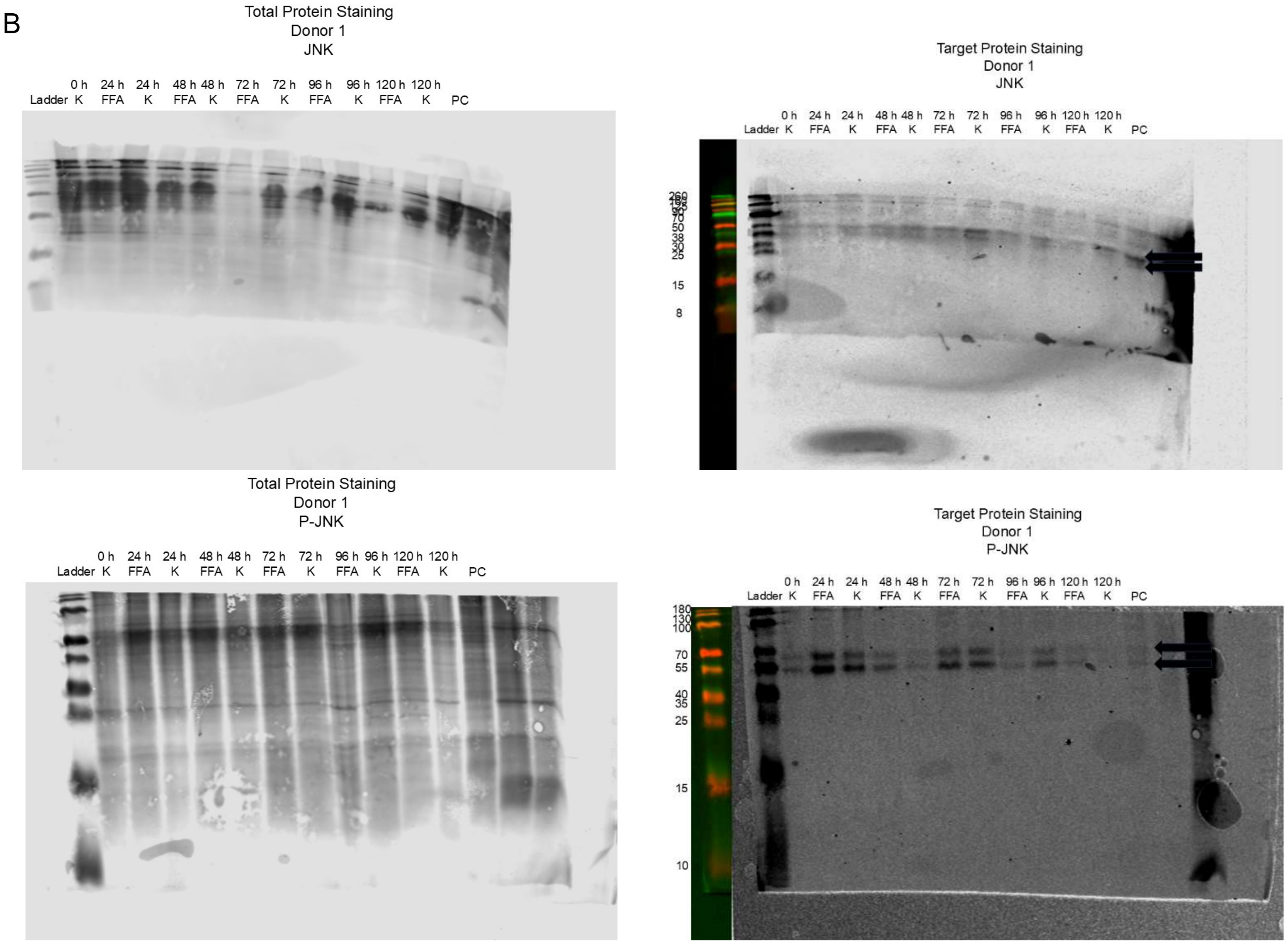

Total Protein Staining
Donor 2
JNK

0 h K | 24 h FFA | 24 h K | 48 h FFA | 48 h K | 72 h FFA | 72 h K | 96 h FFA | 96 h K | 120 h FFA | 120 h K | PC (after Ladder)

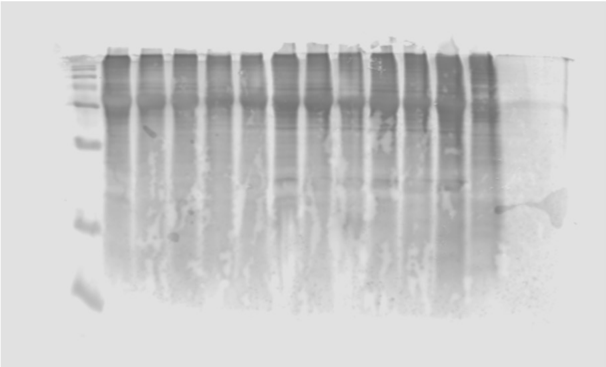

Target Protein Staining
Donor 2
JNK

Ladder | 0 h K | 24 h FFA | 24 h K | 48 h FFA | 48 h K | 72 h FFA | 72 h K | 96 h FFA | 96 h K | 120 h FFA | 120 h K | PC

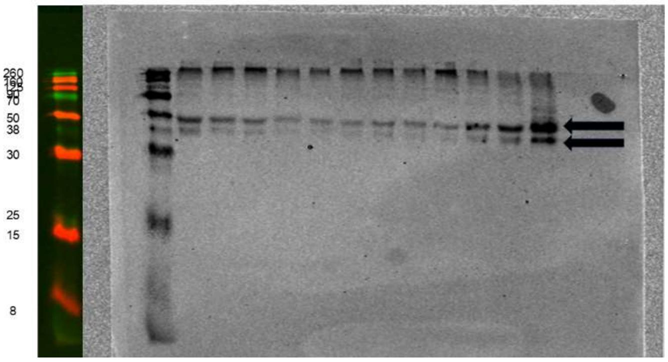

Total Protein Staining
Donor 2
P-JNK

Ladder | 0 h K | 24 h FFA | 24 h K | 48 h FFA | 48 h K | 72 h FFA | 72 h K | 96 h FFA | 96 h K | 120 h FFA | 120 h K | PC

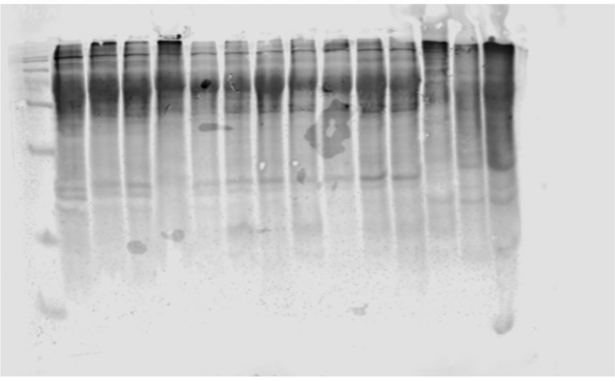

Target Protein Staining
Donor 2
P-JNK

Ladder | 0 h K | 24 h FFA | 24 h K | 48 h FFA | 48 h K | 72 h FFA | 72 h K | 96 h FFA | 96 h K | 120 h FFA | 120 h K | PC

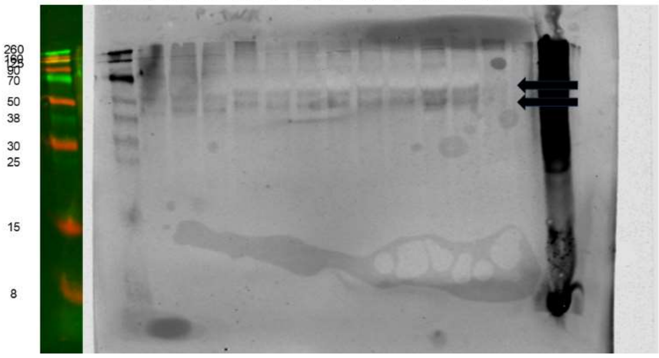

Total Protein Staining
Donor 3
JNK

Ladder | 0 h K | 24 h FFA | 24 h K | 48 h FFA | 48 h K | 72 h FFA | 72 h K | 96 h FFA | 96 h K | 120 h FFA | 120 h K | PC

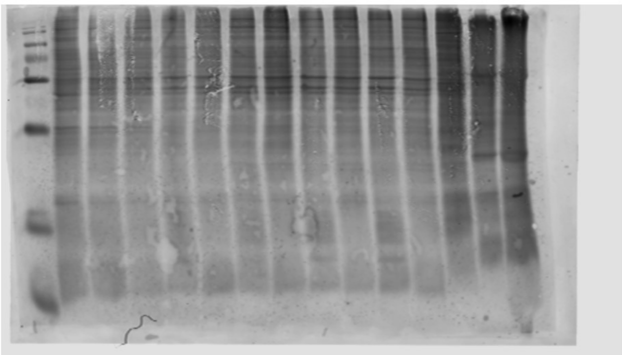

Target Protein Staining
Donor 3
JNK

Ladder | 0 h K | 24 h FFA | 24 h K | 48 h FFA | 48 h K | 72 h FFA | 72 h K | 96 h FFA | 96 h K | 120 h FFA | 120 h K | PC

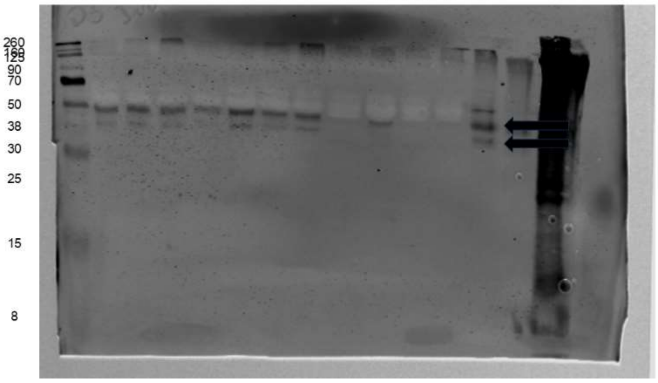

Total Protein Staining
Donor 3
P-JNK

Ladder | 0 h K | 24 h FFA | 24 h K | 48 h FFA | 48 h K | 72 h FFA | 72 h K | 96 h FFA | 96 h K | 120 h FFA | 120 h K | PC

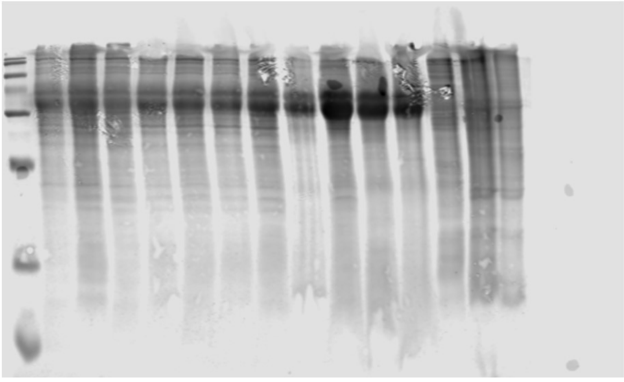

Target Protein Staining
Donor 3
P-JNK

Ladder | 0 h K | 24 h FFA | 24 h K | 48 h FFA | 48 h K | 72 h FFA | 72 h K | 96 h FFA | 96 h K | 120 h FFA | 120 h K | PC

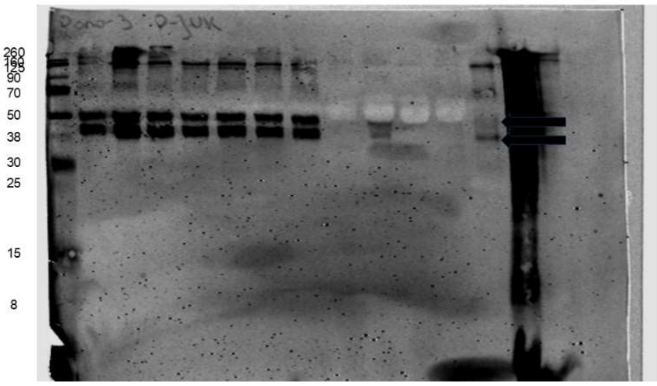

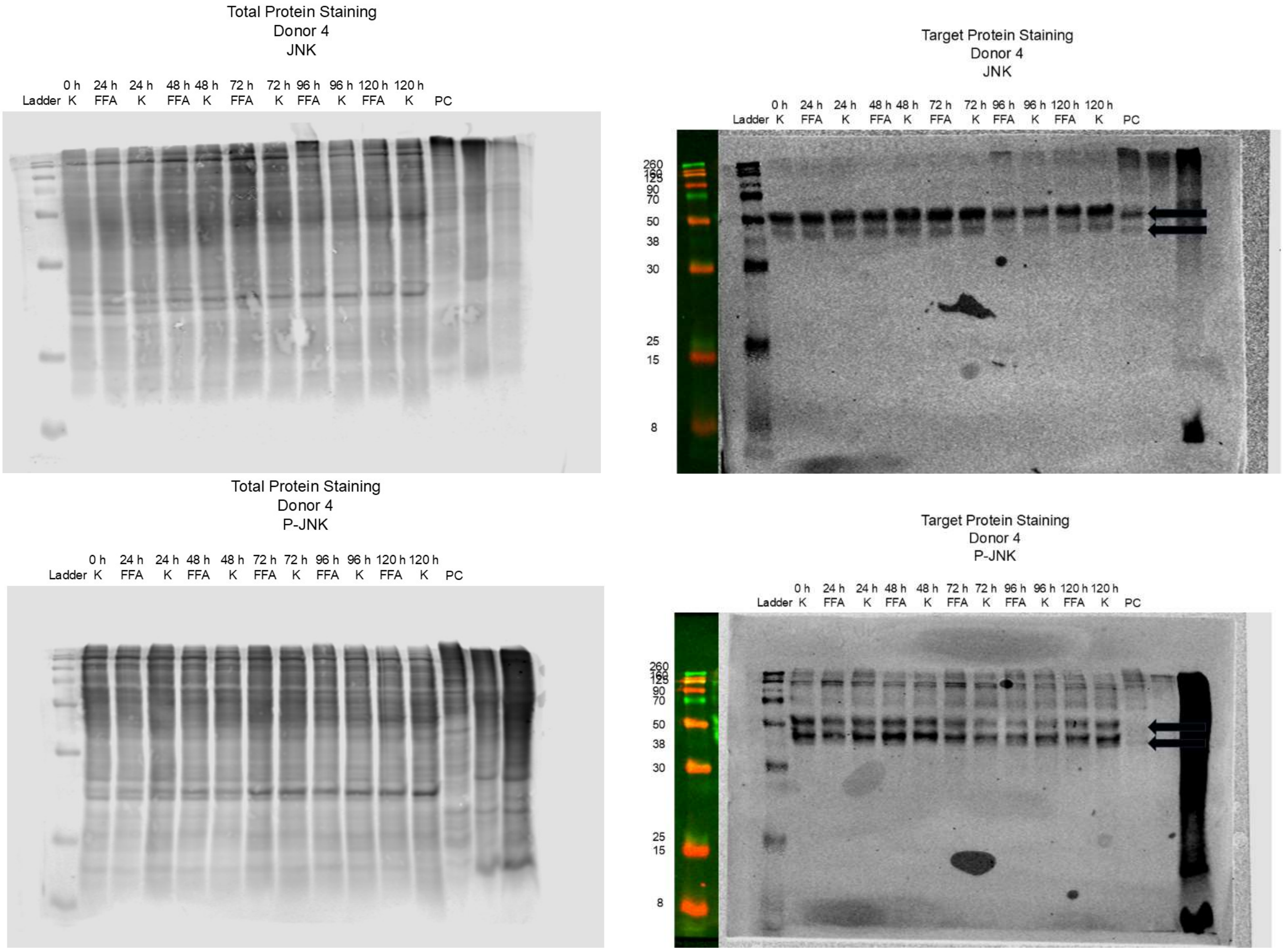

Figure S10: JNK and P-JNK expression

A. Expression of both isoforms (46 kDa and 54 kDa) of P-JNK per JNK in FFA-treated and control group.

B. Total and target protein staining of JNK and P-JNK for each donor

MAP LC3β

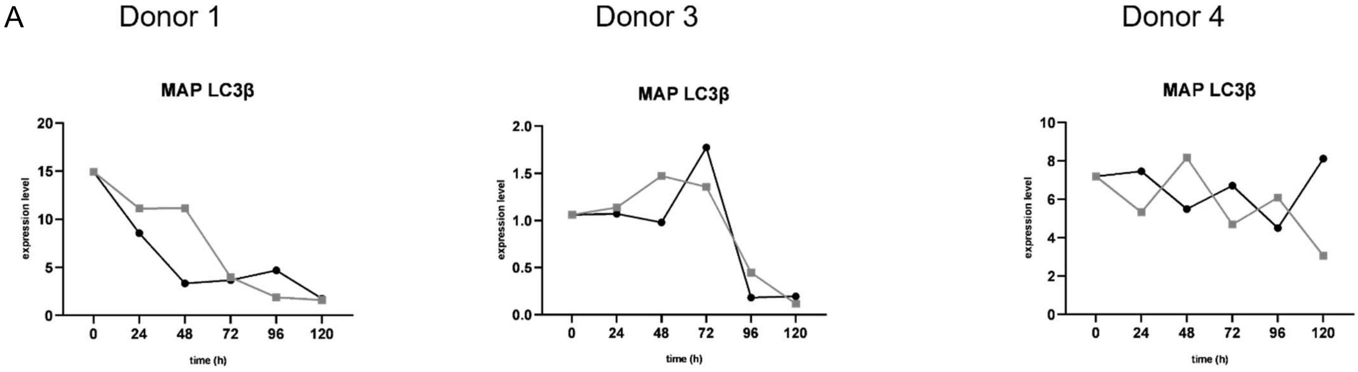

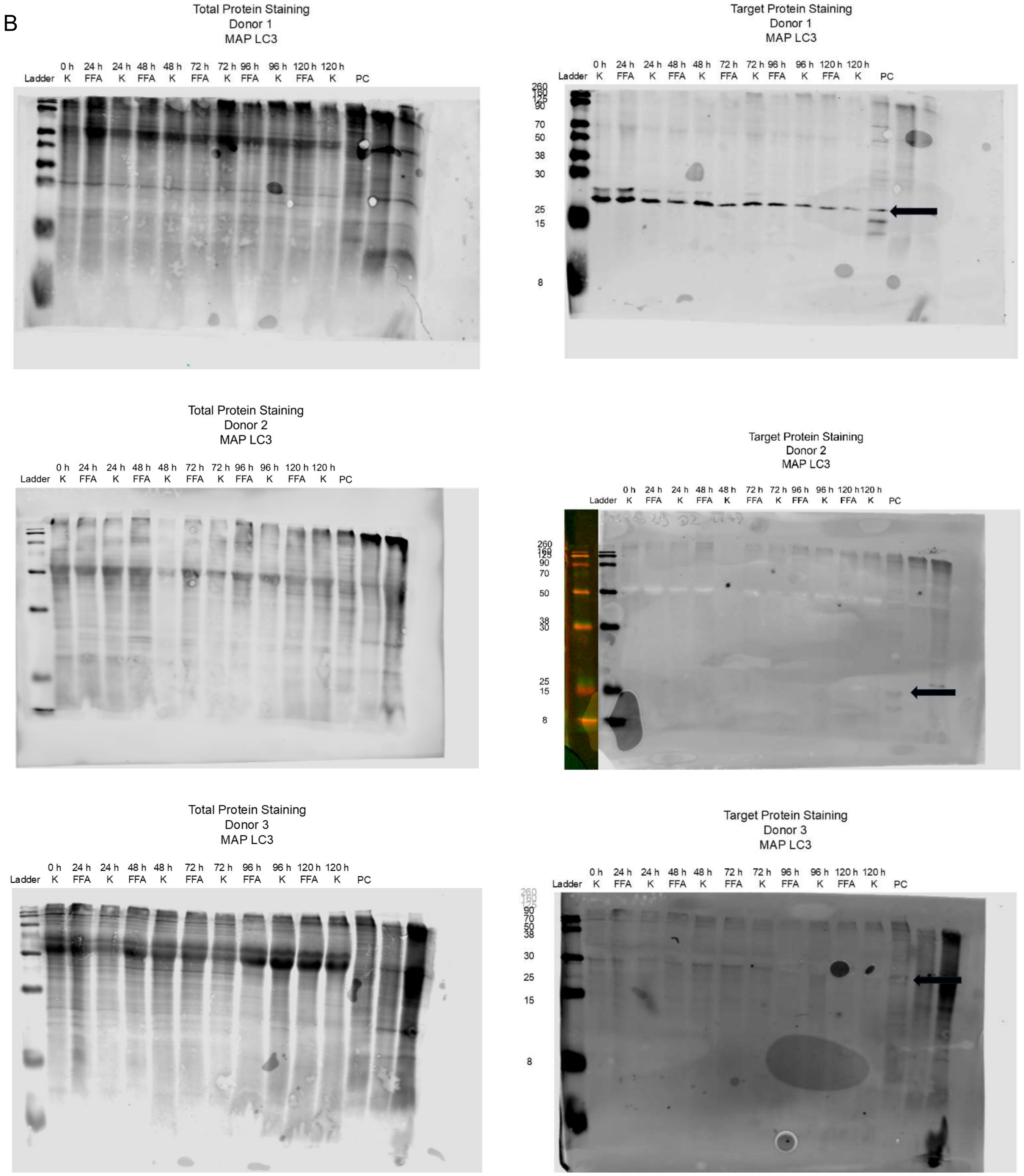
B
Total Protein Staining
Donor 1
MAP LC3
Ladder
0 h K
24 h FFA
24 h K
48 h FFA
48 h K
72 h FFA
72 h K
96 h FFA
96 h K
120 h FFA
120 h K
PC
Target Protein Staining
Donor 1
MAP LC3
260
160
125
90
70
50
38
30
25
15
8
Total Protein Staining
Donor 2
MAP LC3
Target Protein Staining
Donor 2
MAP LC3
Total Protein Staining
Donor 3
MAP LC3
Target Protein Staining
Donor 3
MAP LC3

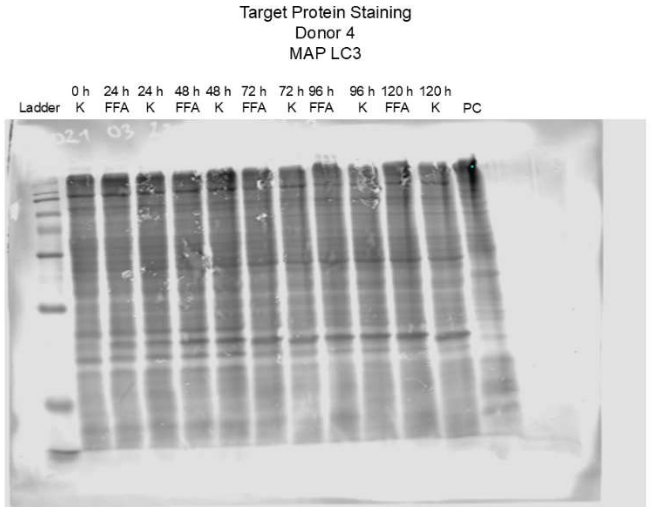

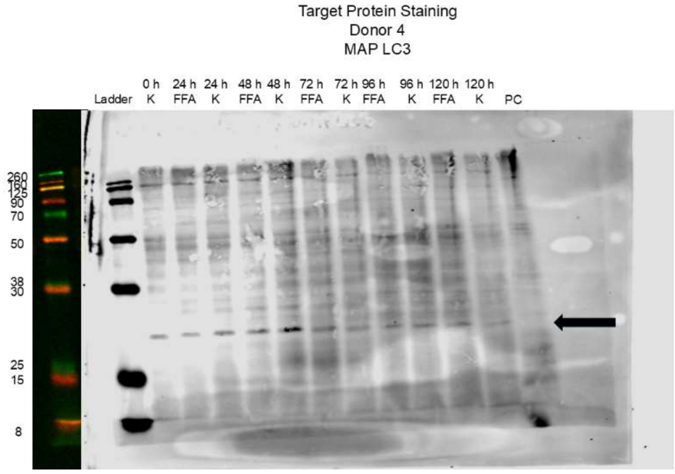

Figure S11: MAP LC3β expression

A. Expression of MAP LC3β in FFA-treated and control group. No expression was observed in donor 2.

B. Total and target protein staining of MAP LC3β for each donor

IRE 1α

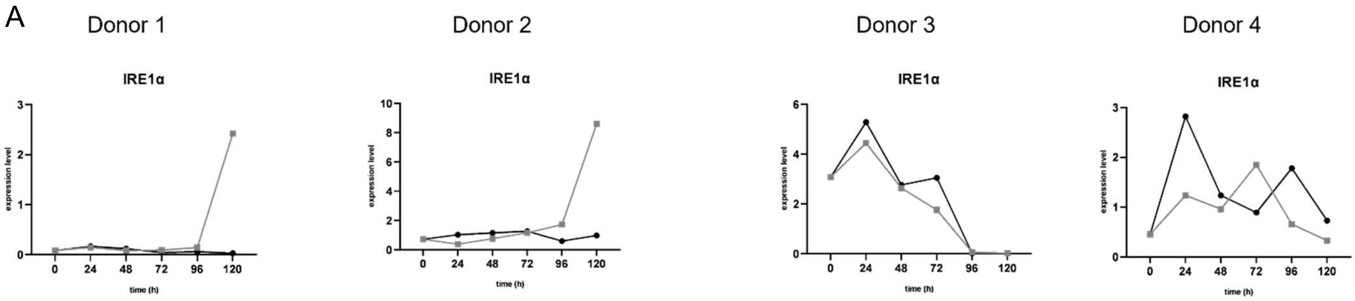

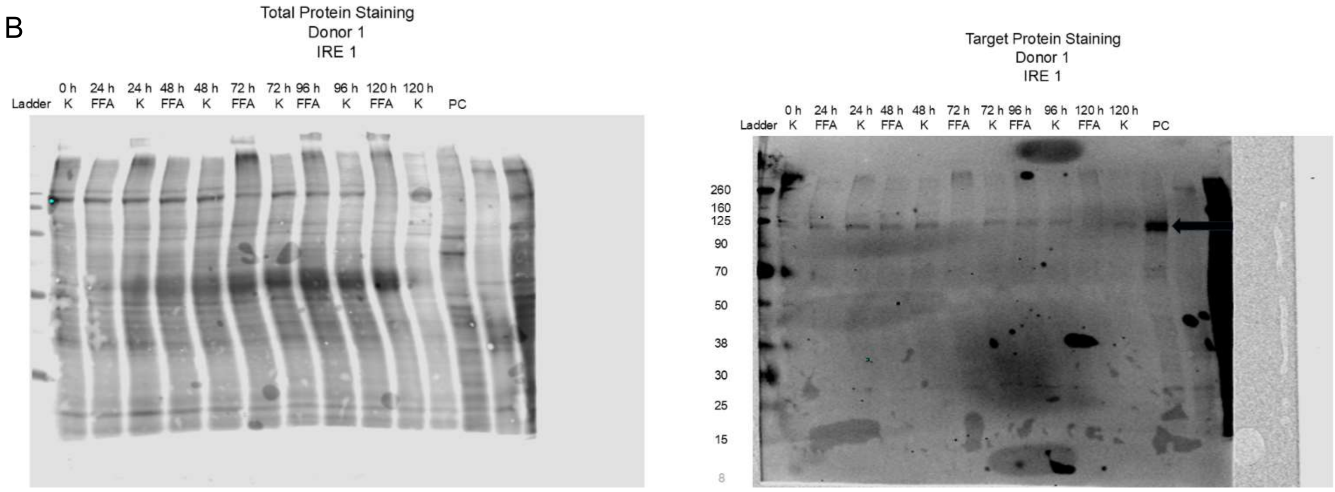

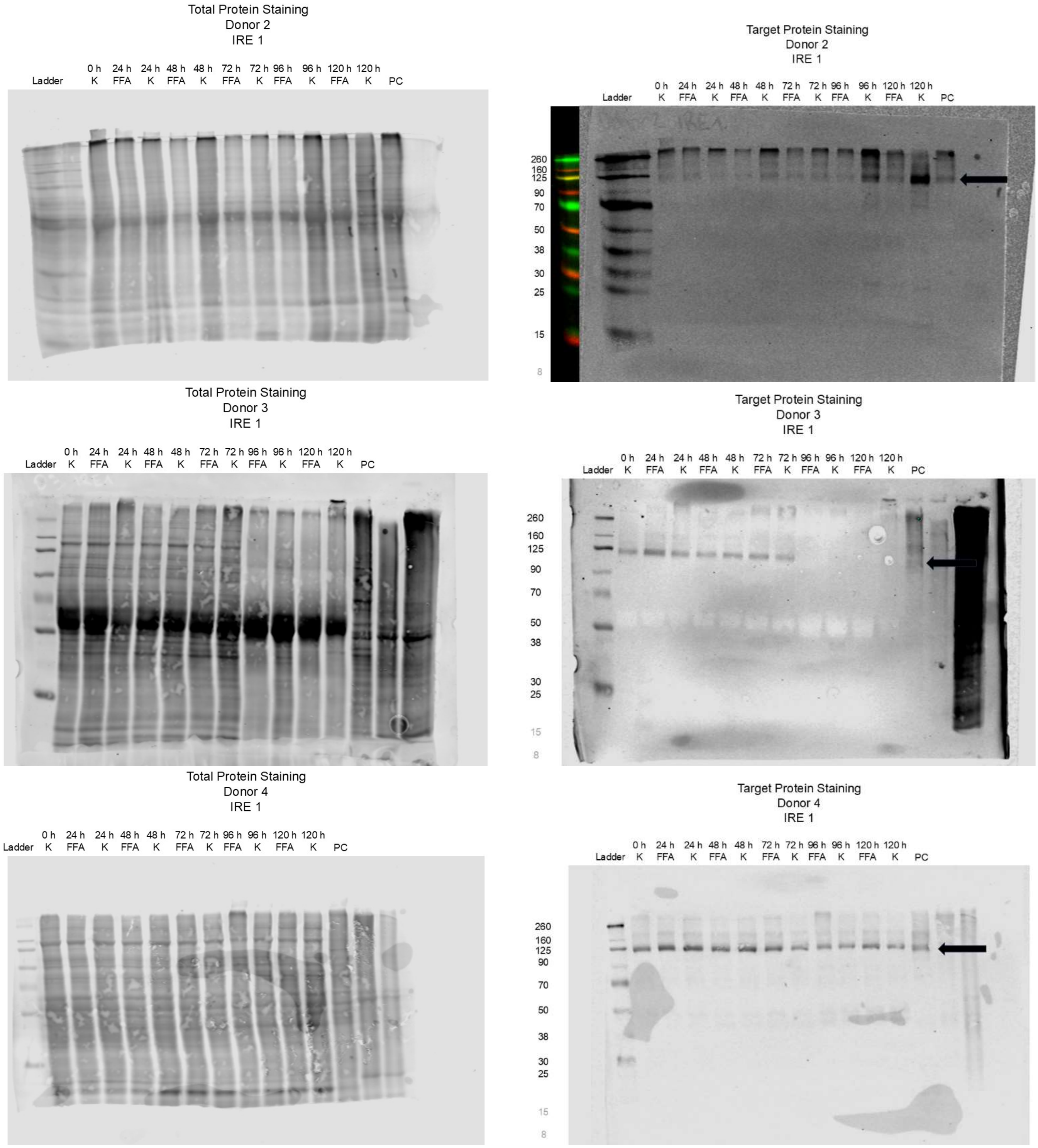

Figure S12: IRE 1α expression

A. Expression of IRE 1α in FFA-treated and control group.

B. Total and target protein staining of IRE 1α for each donor

PPARα

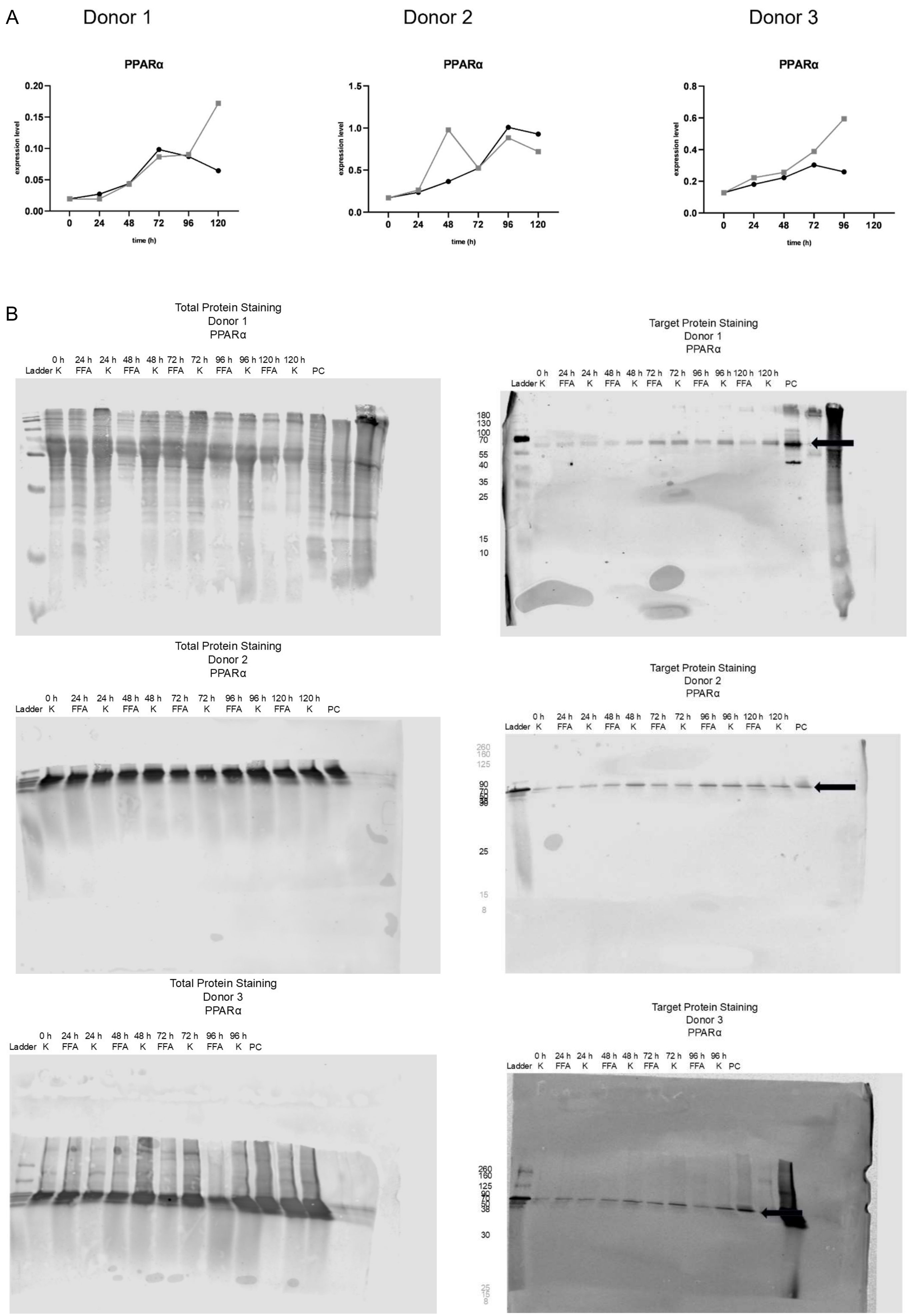

Figure S13: PPARα expression

A. Expression of PPARα in FFA-treated and control group.
B. Total and target protein staining of PPARα for each donor

ATF 6α

A

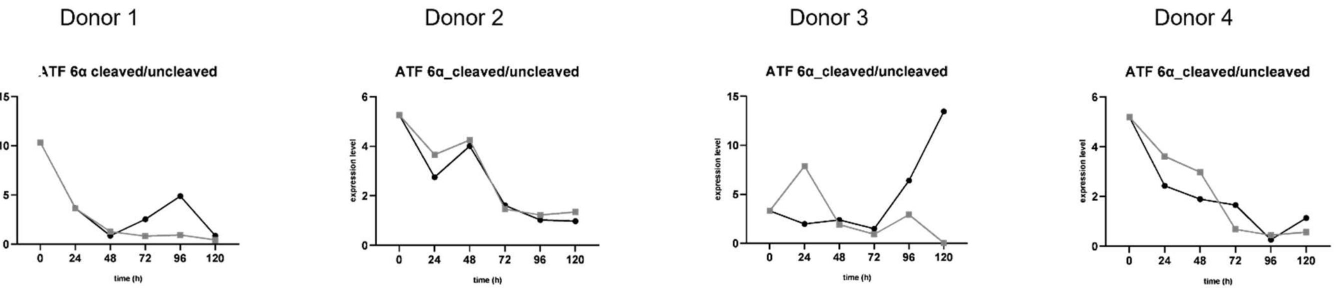

B

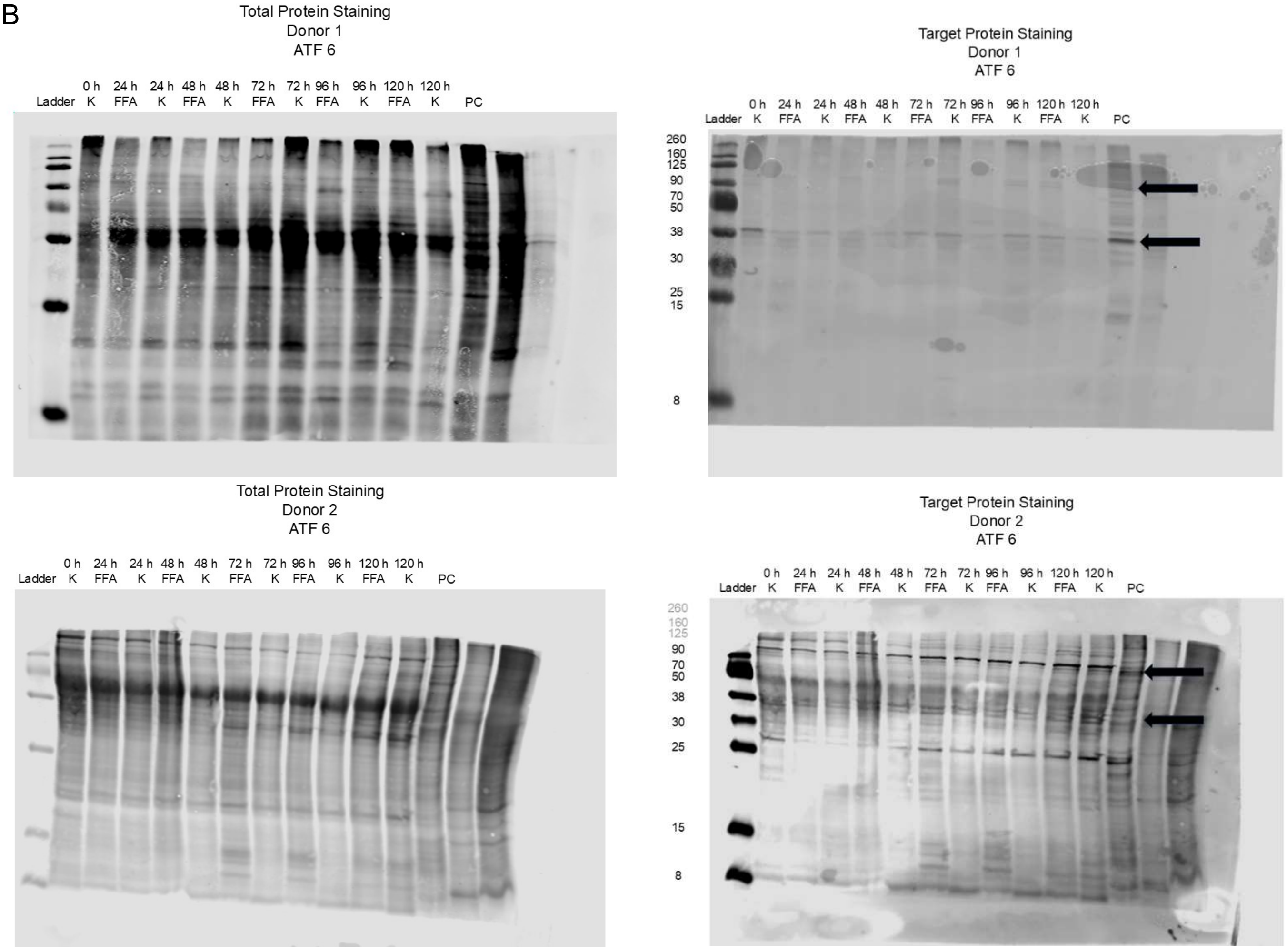

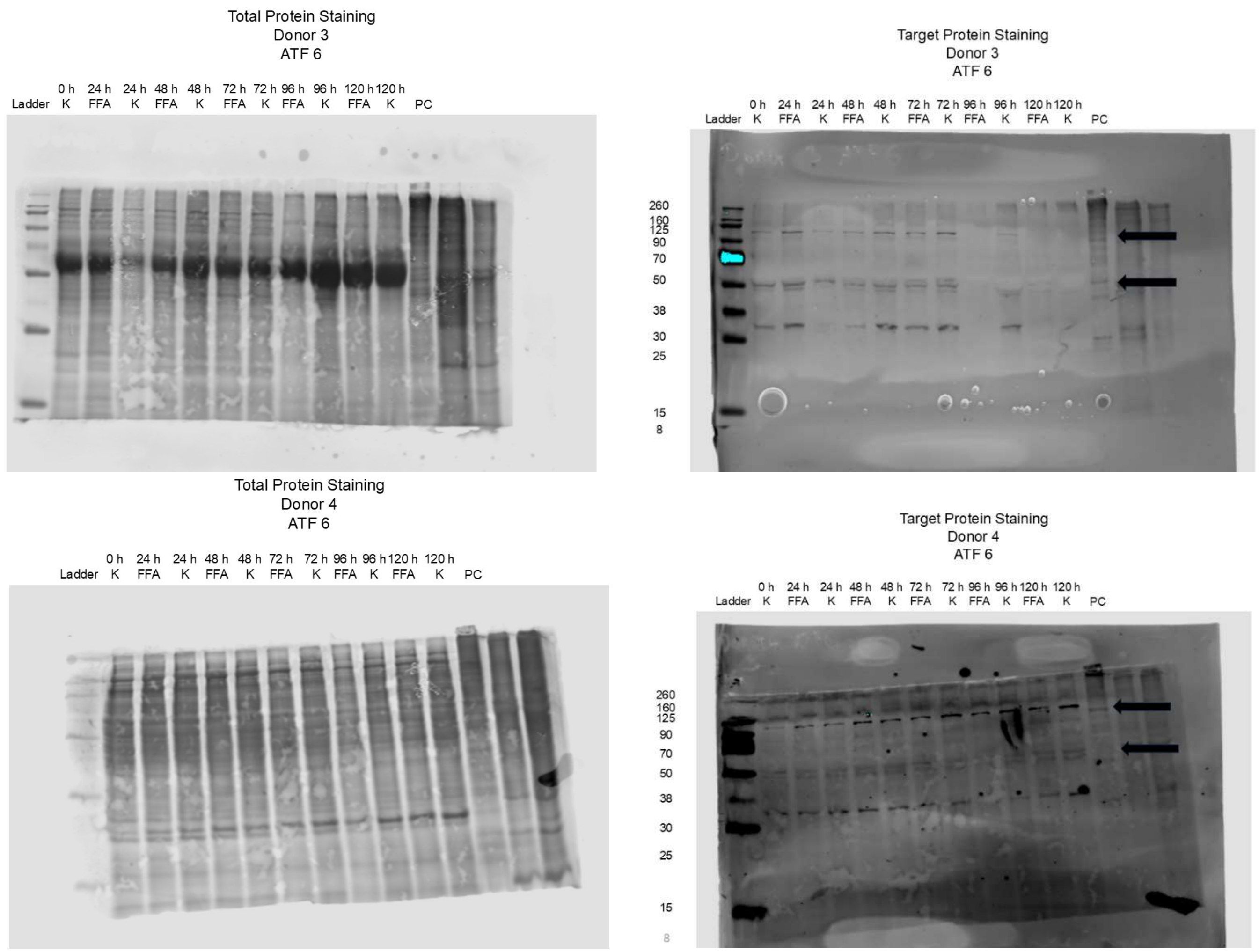

Figure S14: ATF 6α expression

A. Expression of cleaved (90 kDa) per uncleaved (42 kDa) ATF 6α in FFA-treated and control group.

B. Total and target protein staining of ATF 6α for each donor
